# Hybrid Cell Membrane-Functionalized Nanoagents Synergistically Enhance Cuproptosis-Mediated Immunotherapy by Dual Modulation of Glycolytic Metabolism and Tumor Microenvironments

**DOI:** 10.1021/acsnano.5c10671

**Published:** 2025-08-04

**Authors:** Qiang Li, Meng Dang, Ao He, Xiaoye Li, Meng Ding, Zhuo Dai, Yu Zhang, Weijun Xiu, Siyu Wang, Zhusheng Huang, Yongbin Mou, Lianhui Wang, Heng Dong

**Affiliations:** † Nanjing Stomatological Hospital, Affiliated Hospital of Medical School, Institute of Stomatology, 12581 Nanjing University, 30 Zhongyang Road, Nanjing, Jiangsu 210008, China; ‡ Institute for Health Innovation & Technology, Biomedical Engineering Department, 37580 National University of Singapore, 21 Lower Kent Ridge Road, Singapore 119276, Singapore; § Marc and Jennifer Lipschultz Precision Immunology Institute, Icahn School of Medicine at Mount Sinai, 1 Gustave L Levy Pl, New York, New York 10029, United States; ∥ State Key Laboratory for Organic Electronics and Information Displays & Jiangsu Key Laboratory for Biosensors, Institute of Advanced Materials (IAM), Jiangsu National Synergistic Innovation Center for Advanced Materials (SICAM), 12577 Nanjing University of Posts and Telecommunications, 9 Wenyuan Road, Nanjing, Jiangsu 210023, China

**Keywords:** cuproptosis, glycolytic pathway, tumor immunotherapy, nanomedicine, squamous cell carcinoma, melanoma

## Abstract

Glycolytic activity of cancer cells not only reduces their vulnerability to cuproptosis but also heightens the immunosuppressive state of the tumor microenvironment (TME). Our study introduces a nanoplatform called dual-remodeling cuproptosis-inducing agent with hybrid cell membrane coating (DREAM), which is crafted to simultaneously modify the metabolic and immunological landscapes of the TME to enhance cuproptosis-driven immunotherapy. This platform exploits cancer-cell-derived membranes for homologous targeting, enhancing tumor specificity, intratumoral penetration, and intracellular copper delivery. DREAM’s disruption of glycolysis intensifies the copper overload, triggering cuproptosis in cancer cells. Additionally, it alleviates immunosuppression and bolsters immune responses facilitated by the immunogenic cell death from cuproptosis, further potentiated by the immunostimulatory effect of M1 macrophage membranes. The outcome is a pronounced efficacy in curbing tumor growth and distant metastasis in squamous cell carcinoma and also in suppressing melanoma proliferation and lung metastasis. This research underscores the vital interaction among inhibiting glycolysis, enhancing sensitivity to cuproptosis, and activating immune responses, thereby paving a feasible and integrated pathway in cancer immunotherapy.

## Graphical abstract

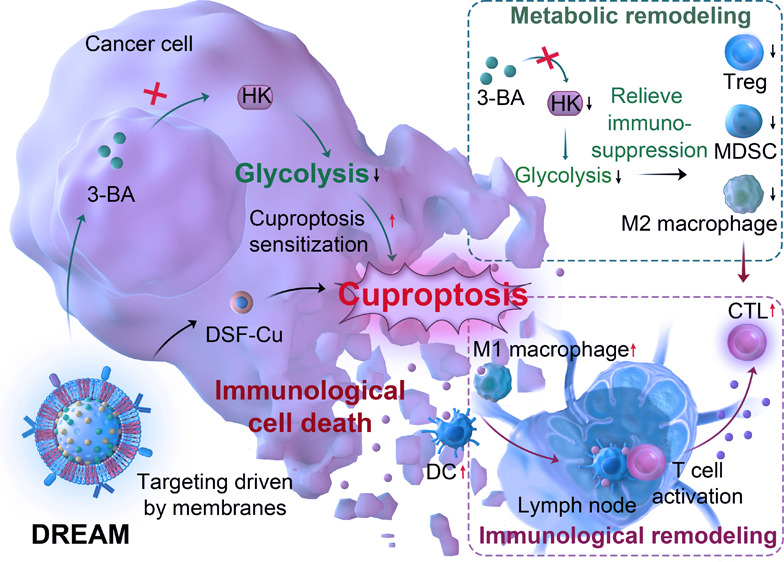

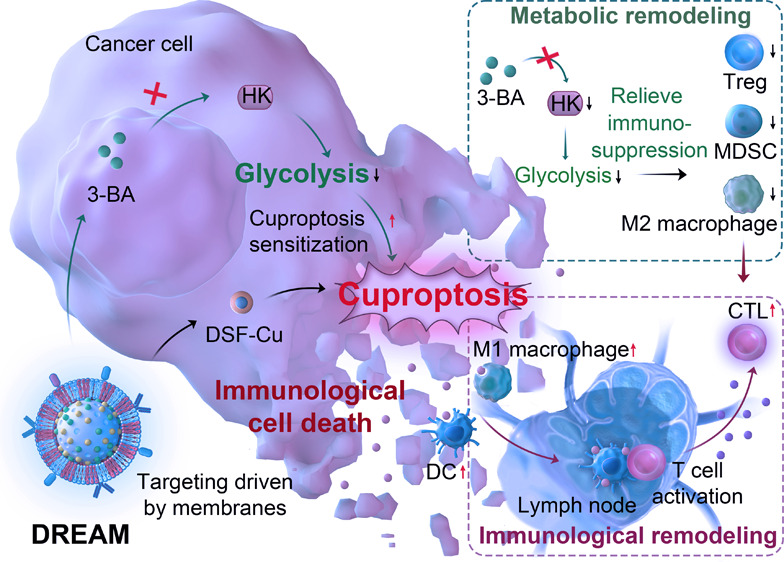

## 1 Introduction

Cuproptosis, a newly identified mechanism of cell death, presents promising opportunities for the development of anticancer therapies.^1^ Triggered by excessive copper accumulation within cells, this process disrupts the mitochondrial tricarboxylic acid (TCA) cycle by oligomerizing lipoacylated proteins, leading to the degradation of iron–sulfur (Fe–S) cluster proteins and inducing lethal proteotoxic stress.^2–4^ Particularly, the susceptibility of cells to cuproptosis is influenced by their metabolic state; cells dependent on mitochondrial respiration are significantly more vulnerable to cuproptosis than those relying on glycolysis, a metabolic pathway often preferred by cancer cells through the “Warburg effect”.^5–7^ Consequently, the inhibition of glycolysis has been explored as a means to not only sensitize cancer cells to conventional therapies but also to augment the effects of immunotherapy by altering cellular metabolism.^8–11^ Moreover, cuproptosis has been identified as a form of immunogenic cell death (ICD), capable of releasing damage-associated molecular patterns (DAMPs) and tumor-associated antigens (TAAs), which enhance the recruitment of dendritic cells (DCs) and cytotoxic T lymphocytes (CTLs).^3,12^ However, the immunosuppressive tumor microenvironment (TME) significantly limits the effectiveness of tumor immunotherapies. Glycolysis by cancer cells not only supports their rapid growth but also induces local nutrient depletion and lactic acid accumulation, further suppressing immune cell function and proliferation.^13,14^ Therefore, targeting glycolysis could transform the TME to promote stronger immune responses against cancer, leveraging the immunogenic potential of cuproptosis-based therapies.

Cell membrane-coated nanoparticles (CNPs), encapsulating synthetic cores within natural cell membranes, represent a significant advance in nanoparticle technology.^15^ These CNPs uniquely emulate complex cellular functions and offer substantial therapeutic potential due to their ability to mimic cellular interactions.^16^ Particularly, cancer cell membranes are known to enhance the circulatory stability of nanoplatforms, evade immune cell phagocytosis, and improve targeting through homotypic binding, which is largely attributed to the diverse antigens present on their surfaces.^17,18^ Moreover, hybrid membranes, produced by fusing cancer and immune cell membranes, exhibit improved immunomodulatory, cancer-targeting, and biological neutralization.^19^ Previous research has expanded to include immune cell membranes such as natural killer cell,^20^ neutrophil cell,^21^ and DCs,^22^ which promote CNPs to activate antigen-specific T cells. M0 phenotype macrophage membranes have also been utilized in biomimetic systems for tumor targeting.^23,24^ The pro-inflammatory M1 phenotype of macrophages significantly enhances antitumor immunity, in stark contrast to the immunosuppressive M2 phenotype typically found in tumor-associated macrophages (TAMs). This distinction has spurred interest in hybridizing M1 macrophage membranes to leverage their pro-inflammatory properties for reshaping the TME, presenting a potentially viable approach for cancer immunotherapy.

Herein, inspired by our findings that glycolysis inhibition could enhance sensitivity to cuproptosis and boost antitumor immune responses, we developed a novel integrated nanoplatform, designated as the dual-remodeling cuproptosis-inducing agent with hybrid cell membrane coating (DREAM), which simultaneously remodeled both metabolic and immunological tumor microenvironments to facilitate effective tumor treatment (Figure 1). The DREAM nanoplatform was composed of Cu-doping mesoporous organosilica nanoparticles that are loaded with disulfiram (DSF) and glycolysis inhibitors, and encapsulated within a hybrid membrane derived from cancer cells and M1 macrophages to avoid nonspecific cuproptosis and enhance the immunogenicity of nanoplatform. The biomimetic design of the DREAM nanoplatform surface enhanced tumor targeting, facilitated penetration into tumor tissue, and promoted the uptake of cancer cells. Inhibition of glycolysis signaling in cancer cells through glycolytic inhibitors significantly amplified the induction of cuproptosis by DREAM. Subsequently, DREAM-based *in situ* nanovaccine induced robust immune responses mediated by cuproptosis through the release of DAMPs and TAAs from dying cancer cells, which were further enhanced by their biomimetic modification of M1 macrophages membrane. This dual-remodeling therapeutic strategy effectively eradicated cancer cells, augmented tumor immunogenicity, and strengthened ICD-mediated immune responses. As a result, DREAM not only demonstrated a substantial therapeutic efficacy against both local tumors and distant metastases of squamous cell carcinoma but also effectively inhibited tumor growth and pulmonary metastasis of melanoma. The DREAM nanoplatform presents a promising strategy for optimizing cancer treatment by inhibiting the glycolytic pathway, sensitizing cancer cells to cuproptosis, and remodeling the TME.

**Figure 1:**
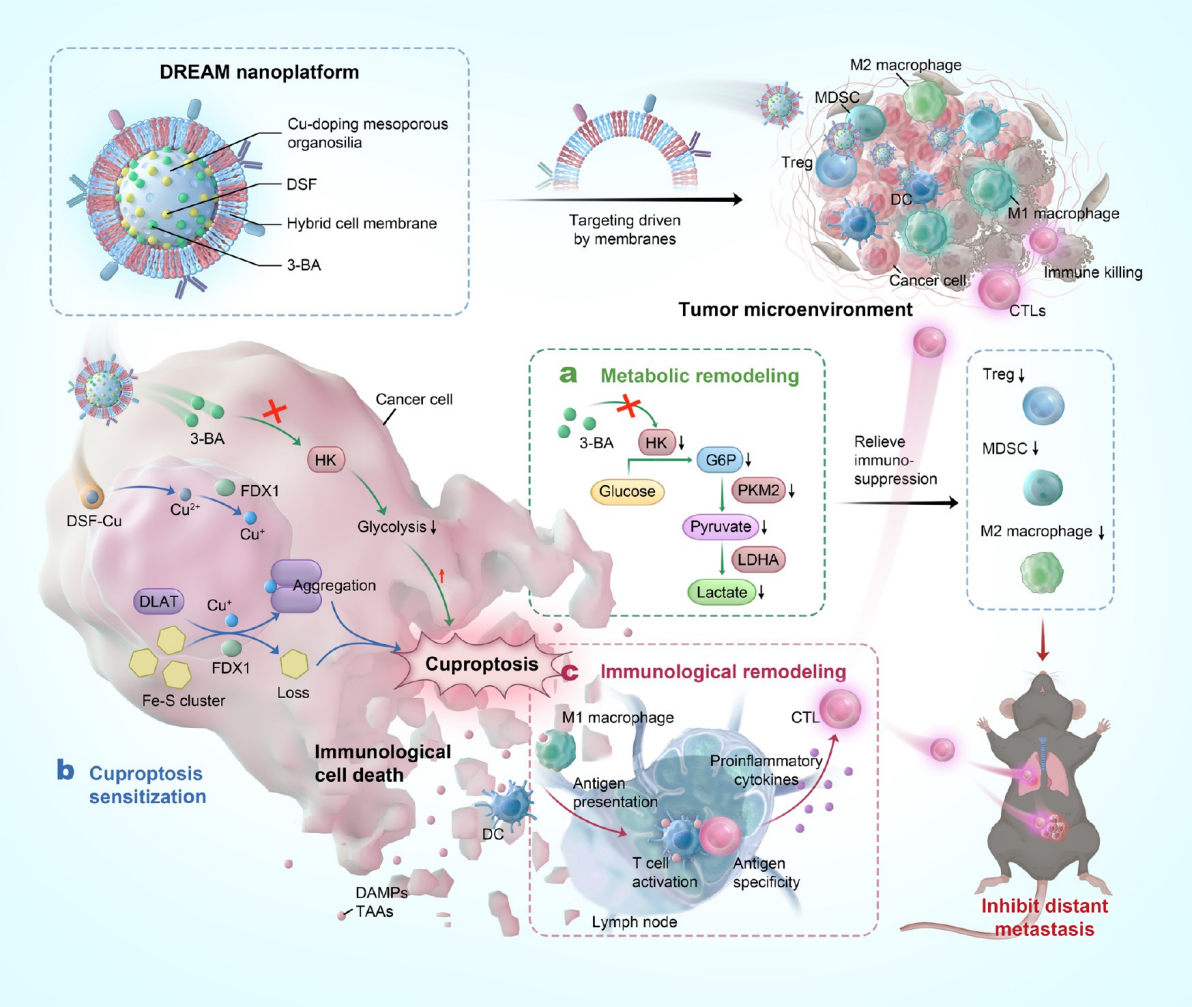
Schematic illustration of the design and mechanism of DREAM for metabolic and immunological dual remodeling to boost cuproptosis-mediated immunotherapy. The DREAM nanoplatform consists of Cu-doped mesoporous organosilica nanoparticles, DSF, 3-bromopyruvic acid (3-BA, glycolysis inhibitor), and a hybrid membrane derived from cancer cells and M1 macrophages. DREAM enhances tumor targeting, facilitates penetration into tumor tissue, and promotes Cu uptake by cancer cells. The inhibition of glycolytic activity significantly amplifies the induction of cuproptosis mediated by DREAM. Subsequently, the DREAM-based *in situ* nanovaccine elicits robust immune responses driven by cuproptosis *via* the release of DAMPs and TAAs, further enhanced by their biomimetic modification using M1 macrophage membranes. This dual-remodeling therapeutic strategy orchestrates the eradication of cancer cells, augmentation of tumor immunogenicity, and activation of immune responses, thus efficiently inhibiting cancer recurrence and metastasis.

## 2 Results and Discussion

### 2.1 Correlation of Cuproptosis, Glycolysis, and Cancer Immunotherapy

While cuproptosis has been investigated across various tumor models, including breast cancer, bladder cancer, glioblastoma multiforme, and colorectal cancer,^25^ its application in head and neck squamous cell carcinoma (HNSCC) remains underexplored, with the underlying mechanisms still not fully elucidated. An analysis of The Cancer Genome Atlas (TCGA) database demonstrated significant disparities in the expression levels of cuproptosis-related genes, including lipoyltransferase 1 (LIPT1), dihydrolipoamide S-succinyltransferase (DLST), and glycine cleavage system protein H (GCSH), between tumor tissues from HNSCC and the corresponding normal tissues (Figure 2a–c). Notably, bioinformatics analyses of single-cell sequencing have revealed that the cuproptosis-related key gene dihydrolipoamide S-acetyltransferase (DLAT) is predominantly expressed in epithelial and keratin cells within HNSCC tumor tissues (Figure 2d,e), indicating that HNSCC may be particularly responsive to therapies targeting cuproptosis. Through immunohistochemical staining analysis of mouse tongue cancer xenografts, we further confirmed that the expression levels of cuproptosis-related markers, ferredoxin 1 (FDX1) and lipoic acid synthetase (LIAS), were significantly elevated in tumors compared to normal tongue tissues (Figure 2f,g). Collectively, these findings suggest that HNSCC may exhibit sensitivity to cuproptosis, indicating that cuproptosis-based therapeutic strategies represent a promising approach for HNSCC treatment.

**Figure 2:**
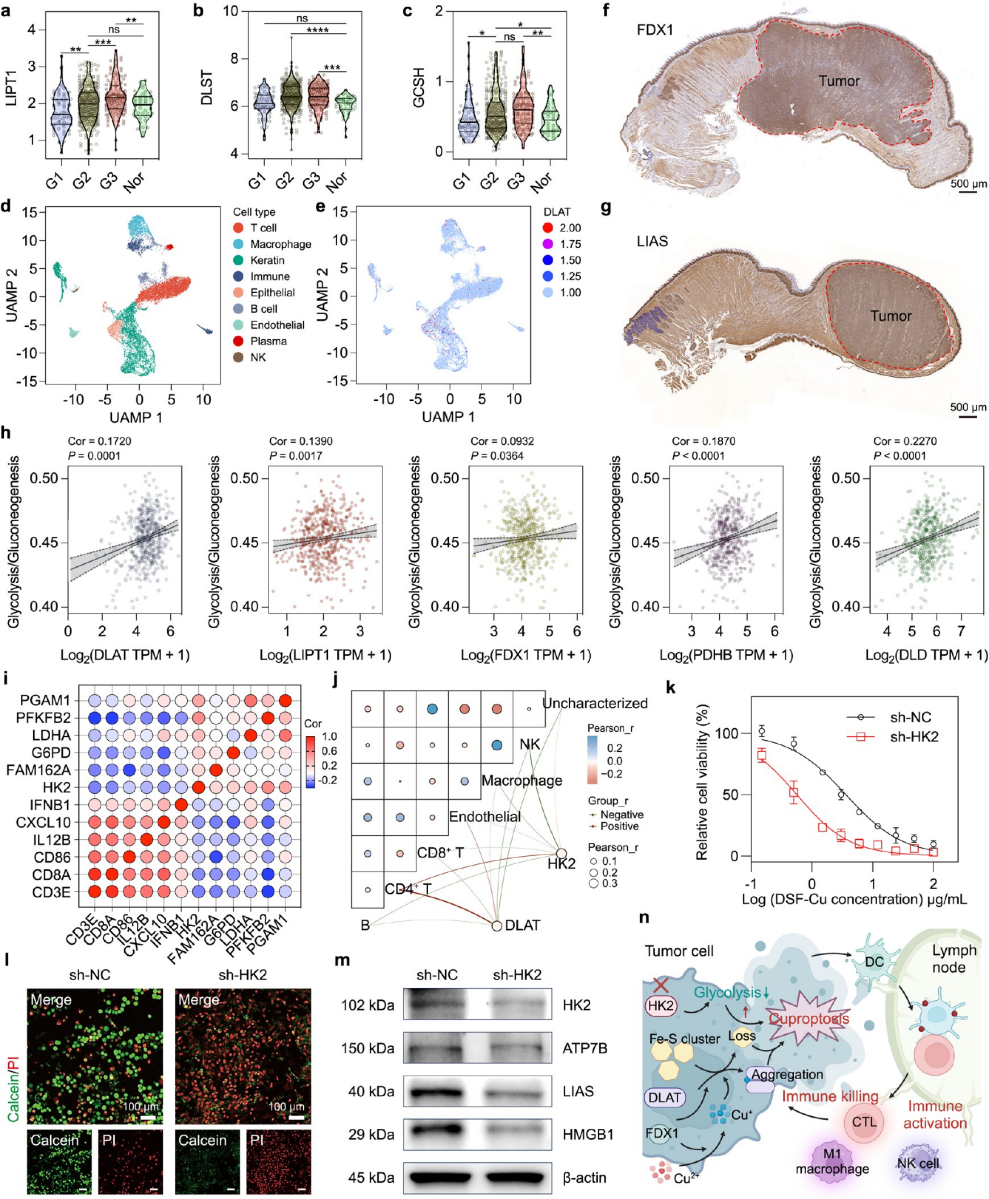
Inhibition of glycolysis is expected to promote tumor cell cuproptosis and enhance immune response. (a–c) The expression distribution of cuproptosis-related genes, including LIPT1 (a), DLST (b), and GCSH (c) in HNSCC tumor tissues and normal (Nor, *n* = 44) tissues. Data were obtained from The Cancer Genome Atlas Program (TCGA) data set. G1, grade 1, well differentiated (low grade) tumors (*n* = 62); G2, grade 2, moderately differentiated (intermediate grade) tumors (*n* = 301); G3, grade 3, poorly differentiated (high grade) tumors (*n* = 119). (d) The t-SNE map of HNSCC single-cell sequencing displays cell clustering with distinct colors representing different cell types. The original data were sourced from the Gene Expression Omnibus (GEO) database with the accession number: GSE172577.^27^ (e) The t-SNE plot visualizing the distribution of DLAT expression across different cells, where varying colors indicate expression abundance. (f, g) Representative FDX1 (f) and LIAS (g) immunohistochemistry of the tongue tumors obtained from C3H/He mice that were orthotopically transplanted with SCC7 cells in the tongues. Scale bars: 500 μm. (h) Spearman correlation analysis between cuproptosis-related genes and the Glycolysis/Gluconeogenesis pathway score. Cor, correlation coefficient. (i) Heatmap illustrating the correlation between glycolysis-related genes and immune activation-related genes in HNSCC tumors. Red indicates a positive correlation, while blue indicates a negative correlation. (j) Correlation analysis between DLAT and HK2 gene expression and immune score in HNSCC tumors. The heatmap illustrates the correlation analysis of the immune score itself, in which red indicates a negative correlation, while blue indicates a positive correlation. Red lines denote positive correlations between gene expression and immune scores, whereas green lines represent negative correlations. (k) Relative cell viability of SCC7-sh-NC and SCC7-sh-HK2 cells after incubation with different concentrations of DSF-Cu for 24 h, as determined by the CCK-8 assay. (l) Confocal laser scanning microscopy (CLSM) images of calcein-AM (green) and PI (red) costained SCC7-sh-NC or SCC7-sh-HK2 cells after incubated with DSF-Cu (1.5 μg/mL). Scale bars: 100 μm. (m) Western blot (WB) for corresponding proteins in the cuproptosis, glycolysis, and ICD pathway as indicated. (n) Schematic illustration of the mechanism by which glycolysis inhibition could promote cuproptosis in tumor cells and enhance immune responses. The statistical significance of differences was tested with the Kruskal–Wallis test (a–c).

To explore the relationship between cuproptosis and glycolysis, we utilized high-throughput sequencing data from the TCGA database for HNSCC mRNA gene expression profiling to compute enrichment scores for the glycolysis/gluconeogenesis pathway.^26^ Remarkably, our analysis revealed a strong correlation among cuproptosis-related genes, specifically DLAT (Correlation coefficient = 0.1720), LIPT1 (Correlation coefficient = 0.1390), FDX1 (Correlation coefficient = 0.0932), pyruvate dehydrogenase E1 subunit β (PDHB, correlation coefficient = 0.1870), and dihydrolipoamide dehydrogenase (DLD, correlation coefficient = 0.2270), within the glycolysis/gluconeogenesis pathway (Figure 1h). Furthermore, genes associated with glycolysis, such as HK2, family with sequence similarity 162 member A (FAM162A), glucose-6-phosphate dehydrogenase (G6PD), lactate dehydrogenase A (LDHA), 6-phosphofructo-2-kinase/fructose-2,6-biphosphatase 2 (PFKFB2), and phospho-glycerate mutase 1 (PGAM1), exhibited a negative correlation with the expression of immune activation-related genes including CD3E, CD8A, CD86, interleukin 12B (IL12B), C-X-C motif chemokine ligand 10 (CXCL10), and interferon β 1 (IFNB1, Figure 2i). This observation reinforces prior findings that glycolysis exacerbates immunosuppressive effects within the TME.^14^ To elucidate the immunological implications of glycolysis and cuproptosis, as well as their potential as therapeutic targets in tumor immunotherapy, we visualized the correlation between immune scores and the key glycolysis gene hexokinase 2 (HK2) alongside the pivotal cuproptosis gene DLAT. The expression of DLAT and HK2 exhibited comparable effects on immune cell infiltration, demonstrating a negative correlation with CD8^+^ T cells and natural killer (NK) cells, while showing a positive correlation with CD4^+^ T cells (Figure 2j), which suggests that the inhibition of glycolysis may enhance sensitivity to cuproptosis and antitumor immune responses.

To further elucidate the mechanistic role of glycolysis inhibition in cuproptosis sensitization and immunological activation, we performed short hairpin RNA (shRNA)-mediated knockdown of the key glycolysis-related gene HK2. SCC7 cells were transfected with lentiviral pLKO.1-shRNA vectors specifically targeting HK2 (sh-HK2) as well as a scrambled control (sh-NC). The FDA-approved small molecule disulfiram (DSF) serves as an effective copper ion carrier, and the complex formed with copper (DSF-Cu) has been reported to be a potent inducer of cuproptosis.^5^ Therefore, we subsequently evaluated the cytotoxic efficiency of DSF-Cu in SCC7-sh-NC and SCC7-sh-HK2 cells. The results showed that the half-maximal inhibitory concentration (IC50) values for SCC7-sh-NC and SCC7-sh-HK2 were 3.61 and 0.56 μg/mL (Figure 2k), respectively. The cell live/dead imaging results further confirmed that DSF-Cu exhibited a higher cytotoxic efficiency against cancer cells with HK2 knockdown (Figure 2l). The results of Western blot analysis revealed that with the inhibition of glycolysis, the expression level of ATPase copper-transporting β (ATP7B) in DSF-Cu-treated SCC7-sh-HK2 cells decreased (Figure 2m), suggesting excessive intracellular copper accumulation in cancer cells. Additionally, LIAS, another marker associated with cuproptosis, exhibited a significant reduction in expression, indicating that glycolysis inhibition further promotes cuproptosis. Furthermore, the release of high mobility group box 1 (HMGB1), a type of DAMP, confirming that cuproptosis-mediated ICD was significantly enhanced upon glycolysis inhibition. These findings indicated that the inhibition of glycolysis could enhance sensitivity to cuproptosis and promotes cuproptosis-mediated ICD, thereby augmenting antitumor immune responses (Figure 2n).

### 2.2 Design of the DREAM Nanoplatform for Efficiently Inducing Cancer Cell Death

Inspired by the significant interconnections among cuproptosis, glycolysis, and immune responses, we designed an integrated biomimetic nanoplatform aimed at enhancing cuproptosis while simultaneously remodeling both the metabolic and immune components of the TME. The cuproptosis-inducing agent (CuIA) was initially synthesized by loading DSF onto a Cu-doping mesoporous organosilica nanoparticle (CuMON). Given that 3-bromopyruvic acid (3-BA) is a potent inhibitor of HK2, the key enzyme responsible for phosphorylating glucose to glucose-6-phosphate in the initial step of glycolysis,^28^ this metabolic remodeling agent (MREA) was subsequently utilized to assemble the dual-remodeling agent (DREA, Figure 3a). To further enhance the tumor-targeting and immune-activating capabilities of the nanoplatform,^29,30^ we isolated the cancer cell membrane along with the membrane from lipopolysaccharide (LPS)-activated M1 macrophages, ultimately encapsulating this hybrid membrane onto the surface of DREA to create DREA with hybrid membrane coated (DREAM).

**Figure 3:**
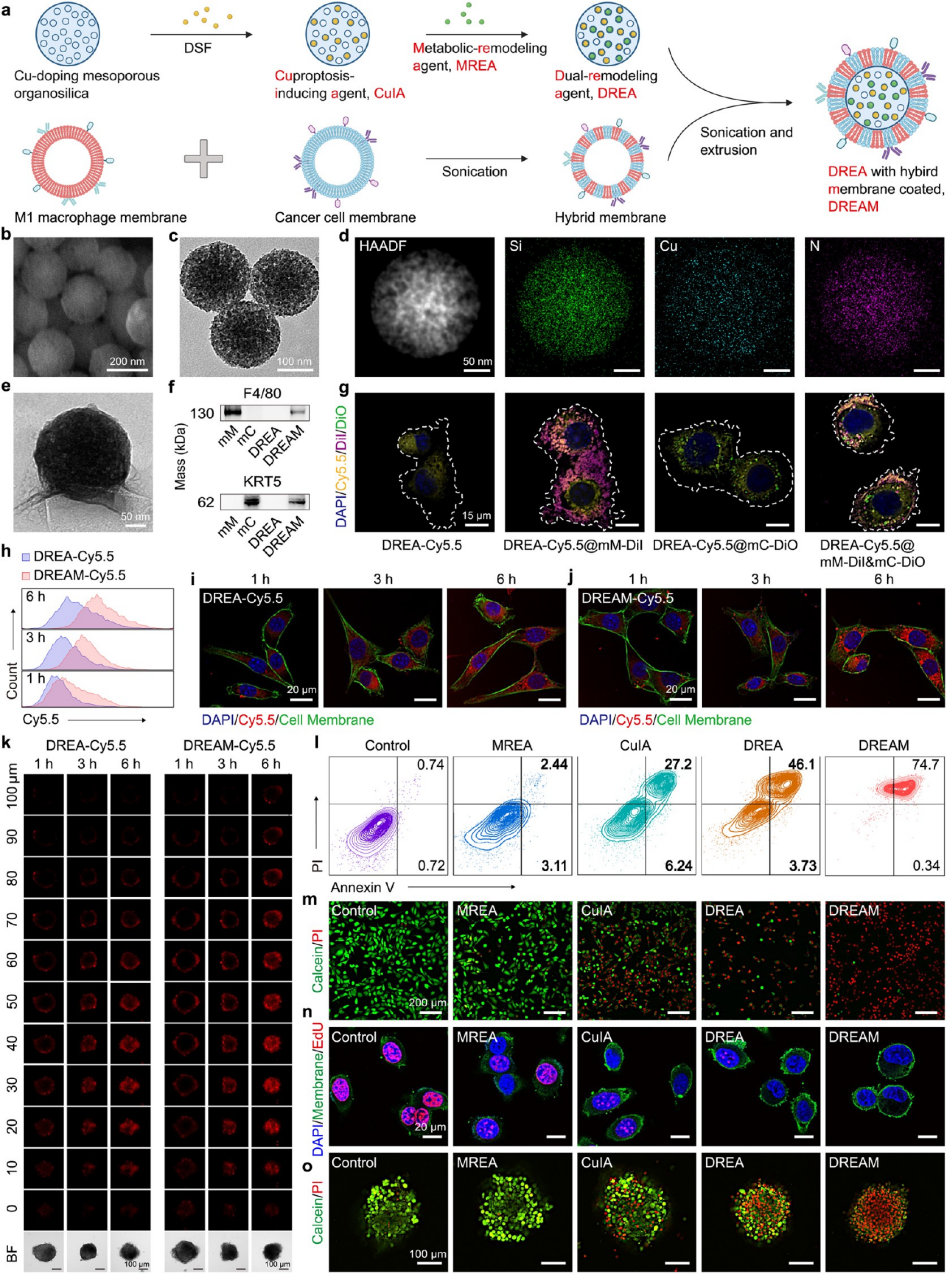
Design of the DREAM nanoplatform for efficiently inducing cancer cell death. (a) Schematic diagram depicting the design of the DREAM nanoplatform. (b) Scanning electron microscopy (SEM) image of DREA. Scale bar, 200 nm. (c) Transmission electron microscope (TEM) image of DREA. Scale bar: 100 nm. (d) High-angle annular dark-field (HAADF) and elemental mapping images of DREA. Scale bars: 50 nm. (e) TEM image of DREAM. Scale bar, 50 nm. (f) WB analysis was performed for F4/80 and cytokeratin 5 (KRT5), which serve as markers for M1 macrophage membranes and SCC7 cancer cell membranes, respectively. Protein samples were obtained from the M1-macrophage membrane (mM), cancer cell membrane (mC), DREA, and DREAM. (g) CLSM images of SCC7 cells incubated with DREA-Cy5.5, DREA-Cy5.5@mM-DiI, DREA-Cy5.5@mC–DiO, and DREA-Cy5.5@mM-DiI&mC–DiO for 6 h. Yellow, Cy5.5; Green, DiO; Purple, DiI; Blue, DAPI. Scale bars, 15 μm. (h) Representative flow cytometry (FCM) histograms of Cy5.5 fluorescence in SCC7 cancer cells treated with DREA-Cy5.5 or DREAM-Cy5.5 for 1, 3, and 6 h. (i, j) CLSM images of SCC7 cells treated with DREA-Cy5.5 (i) or DREAM-Cy5.5 (j) for 1, 3, and 6 h. Red, Cy5.5; Green, DiO-labeled cell membrane; Blue, DAPI. Scale bars, 20 μm. (k) CLSM images of Cy5.5 fluorescence in SCC7 multicellular spheroids incubated with DREA-Cy5.5 or DREAM-Cy5.5 for 1, 3, and 6 h. Scale bars, 100 μm. (l) Representative FCM plots of apoptotic cancer cells with the treatment of PBS (Control), MREA, CuIA, DREA, or DREAM for 24 h as determined by using an annexin V-FITC/PI apoptosis assay kit. (m) CLSM images of calcein-AM (green) and PI (red) costained SCC7 cells with different treatments. Scale bars: 200 μm. (n) CLSM images of SCC7 cells stained with the 5-ethynyl-2′-deoxyuridine (EdU) cell proliferation kit. Red, EdU; Green, DiO-labeled cell membrane; Blue, DAPI. Scale bars: 20 μm. (o) CLSM images of calcein-AM (green) and PI (red) costained SCC7 multicellular spheroids with different treatments. Scale bars, 100 μm.

Images obtained from scanning electron microscopy (SEM) and transmission electron microscopy (TEM) revealed that DREA displayed a quasi-spherical morphology with a consistent diameter of approximately 150 nm (Figures 3b,c and S1). The high-angle annular dark-field scanning transmission electron microscopy (HAADF-STEM) and elemental mapping images clearly displayed the mesoporous architecture of DREA and demonstrated a homogeneous distribution of Cu elements throughout (Figure 3d). Moreover, wide-scan X-ray photoelectron spectroscopy (XPS) analysis identified the presence of Cu, O, Si, and C elements in DREA (Figure S2). Three Cu^2+^ satellite peaks were observed at 962.6, 941.8, and 944.0 eV. The prominent Cu^+^ 2p_3/2_ peak at 935.1 eV and Cu^+^ 2p_1/2_ peak at 954.8 eV revealed the presence of Cu^+^ in DREA. Additionally, the high-resolution O 1s spectrum was divided into two peaks at 532.3 and 530.9 eV, corresponding to the two distinct chemical states of O originating from Si–O and Cu–O bonds; this further substantiates the Cu-doped silica framework present in DREA. The average hydrodynamic diameters of CuMON, CuIA, DREA, and DREAM were measured to be 277.8, 280.1, 283.9, and 294.6 nm (Figure S3), respectively. The corresponding polydispersity indices (PDIs) were determined as 0.116 ± 0.048, 0.114 ± 0.028, 0.1376 ± 0.065, and 0.1310 ± 0.011 (Figure S4); these values indicated that the nanoparticles exhibited a relatively uniform particle size distribution. Furthermore, the ζ-potentials of these nanoparticles were measured to be −25.02 ± 1.34, −3.33 ± 1.07, −10.27 ± 1.39, and −26.45 ± 5.31 mV (Figure S5), respectively, indicating the successful assembly of the DREAM nanoplatform.

The loading efficiency of DSF and MREA was determined to be 26.51 ± 3.12% and 21.82 ± 2.15%, respectively, at an initial feeding ratio of 4:1 (Figure S6). DSF was predominantly loaded physically into the mesoporous channels of CuMON *via* capillary forces, whereas MREA was loaded through coordination interactions between its carboxyl groups and Cu in the CuMON structure as well as hydrogen bonding with Si–OH groups. Moreover, a time-dependent evaluation of the release behavior in response to an acidic tumor microenvironment was then conducted (Figure S6). The release profiles indicated that DSF was effectively released under low-pH conditions, concurrently reacting *in situ* with Cu^2+^ to form DSF-Cu. Additionally, MREA was also released in response to low pH. Under acidic conditions, the dissolution of the CuO framework within the CuMON skeleton compromises carrier stability, thereby releasing Cu^2+^ and liberating DSF, enabling their *in situ* assembly to form DSF-Cu. The release of MREA primarily depends on the protonation effect induced by the acidic environment, which weakens its hydrogen bonding with Si–OH groups and its coordination interactions with Cu^2+^, thus promoting dissociation and release from the carrier.

Cancer cell membranes could serve as a potent source of antigens, eliciting a robust immunostimulatory response and facilitating multiantigenic vaccination.^13,31^ Moreover, hybrid membrane nanovesicles inherently replicate the characteristics of their source cells, thereby conferring combined functionalities such as targeted tumor delivery and remodeling of the tumor immunological microenvironment.^32^ TEM images confirmed the existence of a thin organic layer on the surface of DREAM, in contrast to the uncoated DREA nanospheres, thereby indicating the successful deposition of the hybrid membranes (Figure 3e). The successful encapsulation of the hybrid membrane was also evidenced by the increased hydrodynamic diameter and decreased ζ-potential of DREAM (Figures S3 and S5). The retention of membrane protein fractions from the fusion membrane was further confirmed through SDS-PAGE and Western blot analysis (Figures 3f and S7), indicating that the DREAM nanoplatform biochemically mimics macrophages and cancer cells, thereby holding promise for tumor targeting and reprogramming of the immune microenvironment.

To investigate the internal uptake of nanoparticles coated with the hybrid cell membranes in cancer cells, DREA was labeled with Cy5.5 to produce DREA-Cy5.5, and subsequently coated with DiI-labeled M1-macrophage membrane (mM-DiI) and/or DiO-labeled cancer cell membrane (mC–DiO). Confocal laser scanning microscopy (CLSM) images of cancer cells treated with DREA-Cy5.5@mM-DiI&mC–DiO revealed heightened Cy5.5 fluorescence intensity and increased fluorescence colocalization (Figure 3g), indicating successful encapsulation of DREA by the hybrid cell membrane and enhanced nanoparticle internalization by cancer cells. Moreover, DREA-Cy5.5 and DREA-Cy5.5 encapsulated within hybrid membranes (DREAM-Cy5.5) were synthesized to further explore their potential advantages in tumor cell uptake and tissue penetration. Flow cytometry (FCM) and CLSM observations collectively demonstrated that prolonged incubation led to a significant increase in the uptake of DREAM-Cy5.5 by cancer cells, surpassing that of nonencapsulated DREA-Cy5.5 (Figures 3h–j and S8), indicating a substantial improvement in drug delivery facilitated by the hybrid membrane biomimetic nanocarriers. The three-dimensional (3D) multicellular spheroids (MCSs) of SCC7 cells were cultured to investigate tumor penetration, and the findings suggested that DREAM-Cy5.5 demonstrated enhanced penetration into the MCSs compared to DREA-Cy5.5 (Figure 3k), potentially due to the homologous targeting effect of cancer cell membranes.^33^

The antitumor potential of the DREAM nanoplatform was then assessed using Annexin V-FITC/PI apoptosis assay. The results revealed that the apoptosis rate of SCC7 cells treated with DREAM was 72.81 ± 2.14% (Figures 3l and S9), significantly higher than that of the DREA (45.95 ± 8.42%), CuIA (29.76 ± 3.78%), and MREA (6.44 ± 0.84%) groups, as supported by the cell live/dead imaging results (Figure 3m). The results of 5-ethynyl-2′-deoxyuridine (EdU) cell proliferation detection assay showed that only 0.53 ± 0.12% of cancer cells had proliferative capacity after DREAM treatment (Figures 3n and S10), while 7.38 ± 0.81, 24.57 ± 0.98, and 46.77 ± 4.30% of cancer cells had proliferative capacity in the DREA, CuIA, and MREA groups, respectively. Moreover, treatment with DREAM led to a reduction in the number of surviving cells (green fluorescence) and an increase in the number of dead cells (red fluorescence) within SCC7 MCSs (Figure 3o), revealing the superior tumor tissue penetration and antitumor efficacy of DREAM.

### 2.3 DREAM Inhibited Glycolysis, Augmented Cuproptosis, and Enhanced Immunological Cell Death in Cancer Cells

To elucidate the precise mechanism underlying cancer cell death triggered by the DREAM nanoplatform, RNA sequencing (RNA-Seq) analysis was initially conducted to comprehensively profile the transcriptome of cancer cells subjected to distinct treatment regimens. The transcriptional profiles of SCC7 treated with DREAM exhibited distinct characteristics compared with other treatment groups (Figure S11). In comparison to control SCC7 cells, a total of 5432 differentially expressed genes (DEGs) were identified in DREAM-treated SCC7 cells, with 2148 DEGs upregulated and 3284 DEGs downregulated (Figures 4a, S12, and S13). Moreover, compared to cells treated with DREA, cells treated with DREAM had 867 upregulated DEGs and 66 downregulated DEGs. As depicted in Figure 4b, the heatmap illustrated significant alterations in the differential expression profile of genes associated with cuproptosis (*Dlat*, *Pdhb*, and *Gcsh*), glycolysis inhibition (*e.g*., *Pfkfb2*, *Fam162a*, *Gnpda2*, *Insr*, *Ldhb*, *Pdk1*, and *Pgam1*), and induction of immunogenic cell death (ICD; *e.g*., *Tnf*, *Cxcl1*, *Bax*, *Ccl25*, and *Nlrp3*).

**Figure 4:**
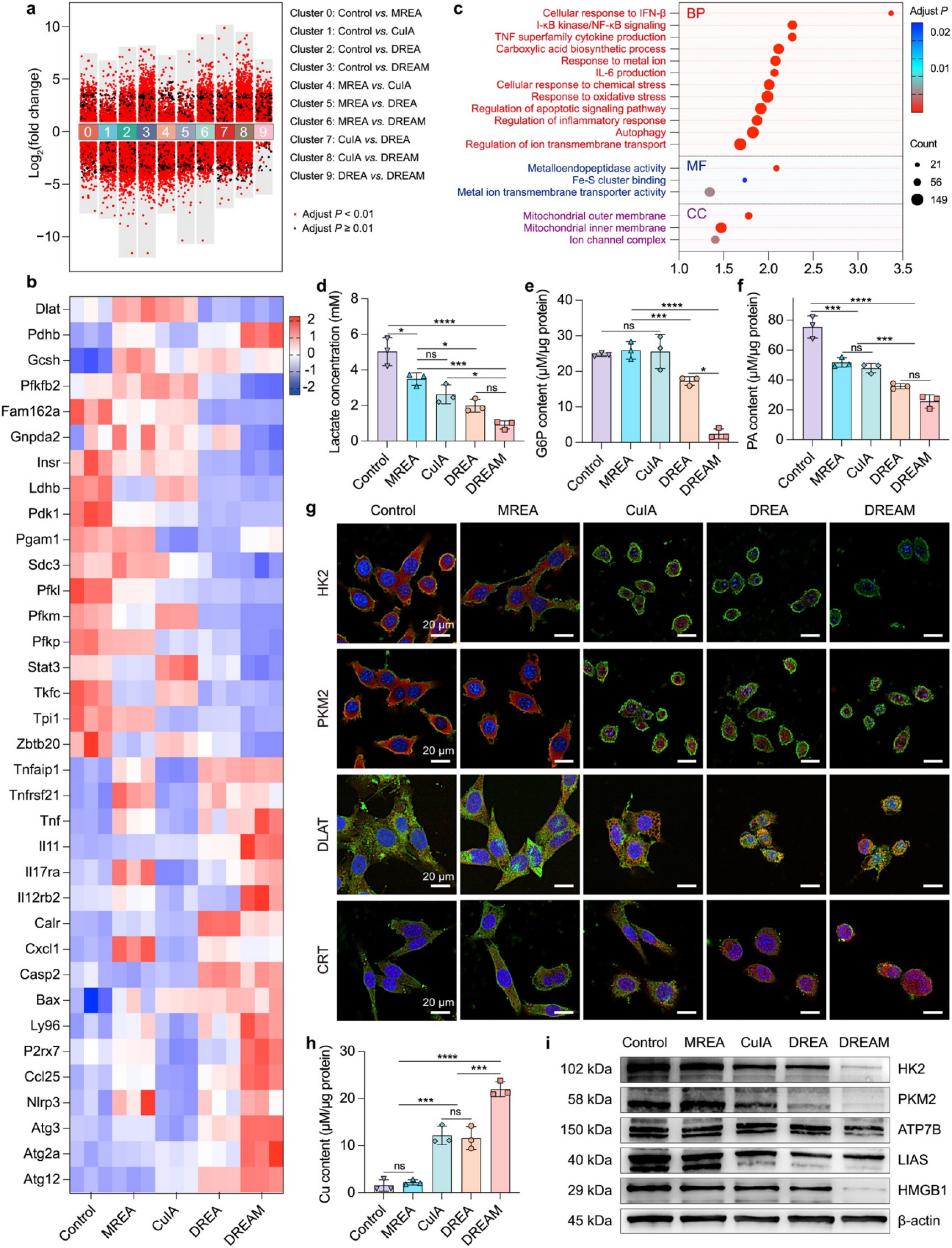
DREAM nanoplatform inhibited glycolysis, increased cuproptosis sensitivity, and promoted immunogenic cell death. (a) Multiple-group volcano plot showing differentially expressed gene (DEG) profiles for each comparison cluster. (b) Heatmap showing the expression profile related to DEGs of cuproptosis, glycolysis, and ICD in cancer cells treated with PBS (Control), MREA, CuIA, DREA, or DREAM. (c) Gene Ontology (GO) enrichment analyses of the DEGs between PBS- and DREAM-treated cancer cells. The dot size represents the number of DEGs associated with each specific term, and the color indicates the level of statistical significance. BP, biological process; CC, cell component; and MF, molecular function. (d) Lactate concentration in the supernatant of cell culture medium of SCC7 cells treated with PBS (Control), MREA, CuIA, DREA, or DREAM for 24 h. (e) Glucose-6-phosphate (G6P) content in the total protein of SCC7 cells treated with PBS, MREA, CuIA, DREA, or DREAM. (f) Pyruvate (PA) content in the total protein of SCC7 cells subjected to various treatments. (g) Representative images of immunofluorescence staining to detect HK2, PKM2, DLAT, and CRT in SCC7 cells subjected to various treatments. Scale bars, 20 μm. (h) Intracellular Cu content in the total protein of SCC7 cells subjected to various treatments. (i) Western blot for corresponding proteins in the cuproptosis, glycolysis, and ICD pathway as indicated. Data are shown as the mean ± s.d. (*n* = 3). The statistical significance of differences was determined using one-way analysis of variance (ANOVA, d–f, and j).

The biological functions of DEGs were linked through Gene Ontology (GO) enrichment analyses, revealing significant associations with cellular response to interferon-β (IFN-β), I-kappaB kinase/nuclear factor kappa-B (NF-κB) signaling pathway, tumor necrosis factor (TNF) superfamily cytokine production, carboxylic acid biosynthetic process, response to metal ions, cellular response to chemical stress, response to oxidative stress, and iron–sulfur cluster binding (Figure 4c). Collectively, transcriptomic analysis revealed that the majority of DEGs in DREAM-treated cancer cells are closely associated with suppressed glycolysis, heightened sensitivity to cuproptosis, and enhanced ICD.

To comprehensively assess the inhibition of glycolysis, we subsequently quantified lactate levels, the ultimate end product of glycolysis, using a lactate assay kit. The treatment with MREA, CuIA, DREA, and DREAM significantly reduced lactate levels in SCC7 cells (Figure 4d), likely due to the inhibition of glycolysis mediated by MREA. Notably, the DREAM nanoplatform demonstrated the most pronounced reduction in lactate expression levels in cancer cells, suggesting that DREAM effectively inhibits glycolysis. Similarly, the intracellular levels of glucose-6-phosphate (G6P) and pyruvate (PA), two critical intermediates in the glycolytic metabolic pathway, were significantly diminished following DREAM-mediated intervention (Figure 4e,4f). Moreover, as demonstrated by immunofluorescent staining and Western blot assays, the significant inhibitory effect of DREAM on glycolysis was further corroborated by a reduction in the expression levels of HK2 and pyruvate kinase isozyme type M2 (PKM2, Figures 4g,4i, S14, and S15).

An adequate concentration of intracellular copper is crucial for the initiation of cuproptosis; however, tumor cells are incapable of maintaining elevated levels of free copper due to their tightly regulated metabolic pathways.^5,34^ The intracellular Cu levels were assessed following various treatments at the 24 h mark, revealing that the DREAM group exhibited the highest Cu concentration, approximately 1.81- and 1.90-fold greater than those in the CuIA and DREA groups, respectively (Figure 4h). In addition to the enhanced cellular uptake observed in Figure 3j, the inhibition of ATP7B by DREAM further resulted in excessive intracellular Cu accumulation in cancer cells treated with DREAM (Figures 4i and S15). The intracellular overload of Cu could bind to the lipoacylated DLAT and induce the heteromerization of DLAT within the mitochondrial tricarboxylic acid cycle, thereby facilitating the degradation of Fe–S cluster proteins and exacerbating proteotoxic stress, ultimately culminating in cuproptosis.^35^ The oligomerization of DLAT was then examined through immunofluorescence imaging. The results indicated that there were no significant foci of DLAT in cancer cells treated with PBS or MREA; however, cells exposed to Cu-based nanoparticles exhibited pronounced DLAT oligomerization, particularly within the DREAM group (Figures 4g and S14). Another cuproptosis-related hallmark, LIAS, was assessed by using WB analysis. In comparison to the elevated expression of LIAS observed in the Control and MREA groups, LIAS levels were significantly reduced in the CuIA, DREA, and DREAM treatment groups, with DREAM exhibiting the most pronounced destabilization effect (Figures 4i and S15). This finding suggests that DREAM could effectively induce cuproptosis in cancer cells.

The association between DREAM-mediated cuproptosis and ICD was further substantiated by examining the release of DAMPs, including calreticulin (CRT) and HMGB1.^36^ The immunofluorescence images indicated a markedly enhanced red fluorescence in the cell membrane of the DREAM group (Figures 4g and S14), suggesting the translocation of CRT from the endoplasmic reticulum to the cell surface. Furthermore, flow cytometry analysis quantitatively demonstrated a significant increase in CRT exposure in SCC7 cells treated with DREAM compared to other groups (Figure S16). Additionally, during ICD, HMGB1 is typically released from the nucleus and subsequently activates TLR-4, thereby facilitating DC maturation.^37^ WB results revealed a substantial reduction in HMGB1 protein levels in SCC7 cells subjected to DREAM treatment (Figure 4i). Notably, the highest concentration of HMGB1 was detected in supernatants from cells treated with DREAM, as quantified by the enzyme-linked immunosorbent assay (ELISA; Figure S17).

The remarkable antitumor efficacy of the DREAM platform further substantiates our hypothesis that the inhibition of the glycolytic signaling pathway by glycolysis inhibitors can reprogram the metabolic state of cancer cells, making them more reliant on mitochondrial respiration and significantly enhancing their sensitivity to cuproptosis.^4^ The potent cuproptosis in cancer cells results in the release of DAMPs, including CRT exposure and HMGB1 release. TAAs released from cuproptosis are internalized more efficiently by DCs due to the “eat me” signal triggered by CRT exposure.^13^ Furthermore, in cells undergoing ICD, HMGB1 is typically released from the nucleus; this released HMGB1 activates Toll-like receptor 4 (TLR-4), thereby promoting DC maturation and enhancing both innate and adaptive immune responses against tumors.^31^ Consequently, the DREAM platform effectively enhances ICD and holds potential for application within a recombinant tumor immune microenvironment.

### 2.4 DREAM Inhibited Primary and Distal Squamous Cell Carcinoma Growth

To further investigate the biodistribution of DREAM nanoplatform encapsulated in the hybrid membrane in tumor-bearing mice, tumor tissues were isolated from the mice 24 h after peritumoral injection of DREA-Cy5.5 or DREAM-Cy5.5 (Figure 5a). The fluorescence intensity of Cy5.5 was notably higher in tumors treated with DREAM-Cy5.5 (Figures 5b and S18). Additionally, fluorescent imaging sections clearly demonstrated significant enrichment of DREAM-Cy5.5 within tumor foci at 24 h postinjection and deeper penetration into the tumor (Figure 5c,5d). In line with findings from *in vitro* studies, these results demonstrated substantial tumor tissue penetration of the DREAM nanoplatform, potentially attributed to adhesion facilitated by the homologous binding of adhesion molecules on fusion membranes, rendering DREAM nanoplatforms more conducive to sustained anticancer effects at tumor sites.

**Figure 5:**
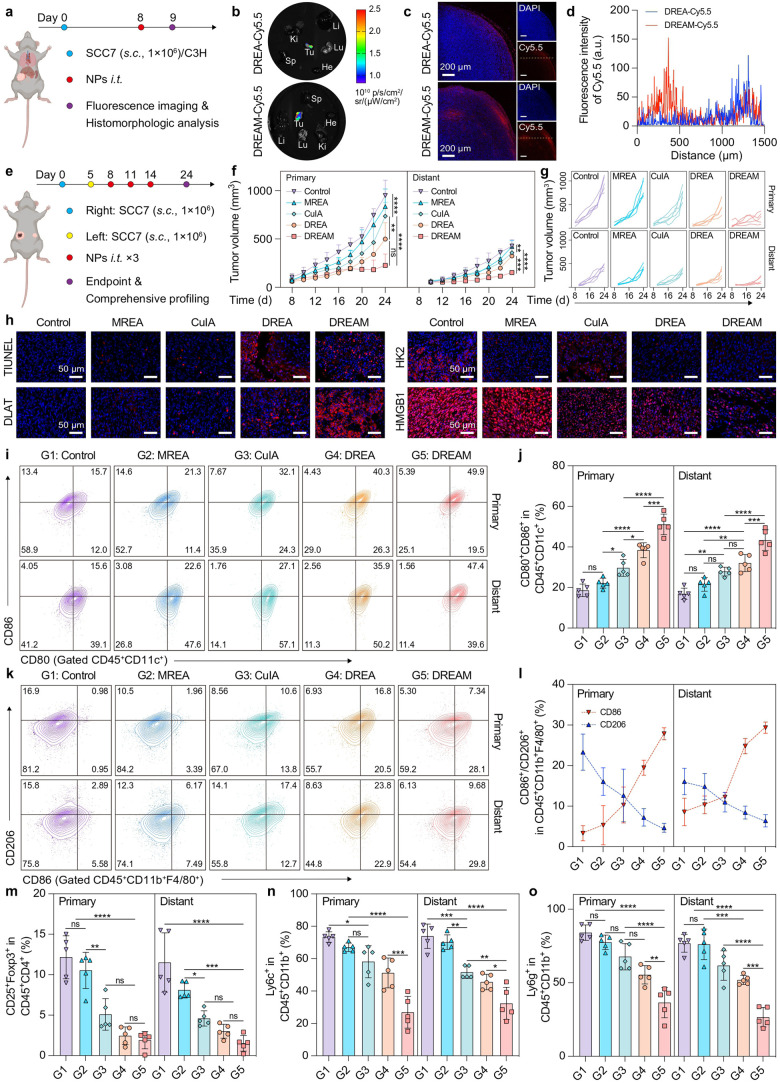
DREAM nanoplatform effectively eradicated SCC7 tumors and induced localized immune responses. (a) Schematic of experimental design for *in vivo* drug distribution in tumor-bearing mice following intratumoral (*i.t*.) injection of DREA-Cy5.5 or DREAM-Cy5.5. (b) Fluorescence images of tumors and major organs obtained from mice 24 h after *i.t*. administration of DREA-Cy5.5 or DREAM-Cy5.5. He, heart; Li, liver; Sp, spleen; Lu, lung; and *K*_i_, kidney. (c) Fluorescence section images of tumors treated with DREA-Cy5.5 or DREAM-Cy5.5. Red, Cy5.5; Blue, DAPI. (d) Relative red fluorescence intensities of the yellow dashed lines are presented in the Cy5.5 panels of the fluorescence images. (e) Schematic illustration of the experimental schedule for the bilateral tumor model, wherein the tumor located on the right side represents the primary tumor under different treatments (G1, PBS; G2, MREA; G3, CuIA; G4, DREA; G5, DREAM), while the tumor on the left side was defined as the distant tumor without any treatment. (f) Average tumor growth curves for primary and distant tumors in SCC7 tumor-bearing mice with different treatments. (g) Tumor growth kinetics for individual mice. (h) Representative histological sections were stained with terminal deoxynucleotidyl transferase dUTP nick end labeling (TUNEL) for apoptosis detection, and immunofluorescent staining using HK2, DLAT, and HMGB1 antibodies. Scale bars: 50 μm. (i, j) Representative FCM plots (i) and the proportions (j) of mature DCs in SCC7 tumors. (k, l) Representative FCM plots (k) and the percentage of M1/M2-type macrophages (l) in tumors. (m–o) Proportions of Tregs (m), M-MDSCs (n), and PMN-MDSCs (o) in SCC7 tumors. Data are shown as the mean ± s.d. (*n* = 5). Statistical significance was calculated using two-way ANOVA (f) and one-way ANOVA (j, m–o).

To investigate the potential of DREAM in suppressing primary and distal tumor growth, we employed bilateral SCC7 tumor-bearing mice as a clinically relevant model for squamous cell carcinoma, which exhibits limited immunogenicity and low response rates to traditional immunotherapies.^38,39^ The bilateral tumor model was established by heterotopically transplanting SCC7 cancer cells into the right flank to form the primary tumor on Day 0, and into the left flank of the same mouse to establish the distant tumor on Day 5 (Figure 5e). Subsequently, intratumoral (*i.t*.) injections of PBS, MREA, CuIA, DREA, or DREAM were administered on Days 8, 11, and 14. The tumor volume of the Control group (with or without NIR irradiation) showed a significant increase, reaching 938.51 ± 158.45 mm^3^ (primary) and 426.52 ± 63.88 mm^3^ (distant) by Day 24 (Figures 5f,g and S19). Remarkably, the DREAM nanoplatform resulted in a significant reduction of primary tumor size, with an average final tumor volume of 199.24 ± 117.52 mm^3^, while DREA only demonstrated moderate inhibition of tumor growth (474.72 ± 169.46 mm^3^). In contrast, treatments with MREA and CuIA exhibited limited antitumor effects on primary tumors, resulting in tumor volumes of 812.86 ± 182.31 and 725.41 ± 143.01 mm^3^, respectively. Moreover, the administration of DREAM demonstrated efficient suppression of both primary and distant tumors (Figure 5f,5g), suggesting an abscopal effect of DREAM-inspired immunotherapy and the potential for DREAM to serve as an immune activator in eliciting systemic immunity. Meanwhile, the DREAM treatment also remarkably prolonged the survival time of tumor-bearing mice compared with the control group (Figures S20). Furthermore, the application of DREAM did not result in a noteworthy decrease in body weight or detectable histological changes in vital organs when compared to the control group (Figures S21 and S22), suggesting the favorable *in vivo* biocompatibility of DREAM.

As shown in Figure 5h, the tumor samples were collected for histological assessment using hematoxylin and eosin (HE) staining as well as terminal deoxynucleotidyl transferase dUTP nick end labeling (TUNEL) staining. Treatment with DREAM resulted in the highest incidence of apoptotic cancer cells, consistent with its potent ability to induce cancer cell death. Immunofluorescence staining of HK2 revealed a significant reduction in its expression in tumor tissues following DREAM treatment, indicating the inhibitory effect of DREAM on glycolysis and the resultant metabolic reprogramming in tumor tissues. The immunofluorescence staining results of DLAT suggested that DREAM-treated tumor tissues underwent cuproptosis, thus confirming the sensitization of cuproptosis by metabolic reprogramming *in vivo*. Furthermore, immunofluorescence images revealed a distinct and significant increase in CRT exposure in tumors subjected to DREAM treatment (Figures 5h and S23), providing evidence of the successful induction of ICD *in vivo*. Moreover, HMGB1, another typical damage-associated molecular pattern molecule, was predominantly localized in the neurons of control SCC7 tumors, whereas a faint fluorescence signal of HMGB1 was observed in the DREAM group (Figure S24), primarily due to the release of HMGB1 following ICD in tumor tissues.

### 2.5 Tumor Microenvironment Immune Remodeling by the DREAM

The exposure of ICD-triggered DAMPs promotes the maturation of DCs, enabling them to present tumor-associated antigens to T lymphocytes and thereby activate systemic antitumor immunity. Moreover, the M1 macrophage membrane on the surface of the DREAM nanoplatform not only exhibits tropism toward tumor sites but also polarizes TAMs into the M1 type, further enhancing antitumor immune activation.^40,41^ Consequently, we subsequently assessed the immune microenvironment reprogramming induced by the DREAM nanoplatform through a comprehensive immunoassay profiling. The proportions of tumor-infiltrating immune cells were initially evaluated in both primary and distant tumors to confirm that the enhanced therapeutic efficacy resulted from the augmentation of systemic immune responses following DREAM-mediated metabolic reprogramming, cuproptosis, and ICD. The FCM analysis revealed a significantly higher maturation ratio of tumor-infiltrating DCs in the TME treated with DREAM (primary, 50.96 ± 5.09%; distant, 43.14 ± 5.11%) compared to the Control group (primary, 18.44 ± 3.08%; distant, 16.72 ± 2.79%), while DREA (primary, 38.24 ± 3.70%; distant, 31.84 ± 4.22%) only showed a moderate induction of DC maturation (Figure 5i,5j). Moreover, a notable increase in M1-like TAMs and a decrease in M2-like TAMs were observed in both the primary and distant tumors following DREAM treatment (Figure 5k,5l), indicating the reprogramming of TAMs toward an antitumor M1 phenotype.

The TME of solid tumors, characterized by immunosuppressive properties, facilitates tumor progression and enables evasion from the immune system, thereby significantly hindering antitumor responses.^42,43^ Subsequently, in addition to M2 TAMs infiltration, we conducted an assessment of the impact of DREAM on the presence of immunosuppressive cells, encompassing regulatory T cells (Tregs) and myeloid-derived suppressor cells (MDSCs).^44^ A noticeable reduction in Tregs was observed in both primary and distant tumors following treatment with the DREAM nanoplatform, as compared with other groups (Figures 5m and S25). Furthermore, a significant decrease in the percentage of monocytic-MDSCs (M-MDSCs, CD45^+^CD11b^+^Ly6c^+^, Figures 5n and S26) was evident in both primary and distant tumors within the DREAM group. Additionally, there was a notable decrease in the proportion of polymorphonuclear-MDSCs (PMN-MDSCs, CD45^+^CD11b^+^Ly6g^+^, Figures 5o and S27), further confirming the mitigation of the immunosuppressive TME through utilization of DREAM.

To comprehensively assess systemic immune activation, the concentrations of secreted cytokines in the serum of mice with SCC7 tumors were determined by using a cytometric bead array. Serum levels of pro-inflammatory cytokines, including IL-1β, IL-6, IL-23, IFN-γ, TNF-α, and CXCL1, were significantly elevated in the DREAM group compared to the other groups (Figures 6a and S28). Moreover, IL-10, a cytokine known to inhibit the antigen-presenting capabilities of DCs, suppresses T cell activation and induces the production of immunosuppressive factors that facilitate tumor immune evasion,^45^ which was observed to have diminished expression in the serum of mice treated with DREAM. A notably higher proportion of mature DCs in the tumor-draining lymph nodes within the DREAM group (Figures 6b and S29), providing further evidence for the systemic immune activation induced by DREAM. As the primary peripheral immune organ in the body, the spleen constitutes approximately 25% of circulating T lymphocytes, which actively participate in cellular immunity against specific tumor cells.^46^ Treatment with DREAM resulted in an elevation of CD8^+^ T cells and a reduction of CD4^+^ T cells in the spleen of tumor-bearing mice (Figure 6c,d), indicating that DREAM effectively elicited cellular immunity. Moreover, the DREAM group demonstrated a significantly higher infiltration of CD8^+^ T cells compared to CD4^+^ T cells within the TME, accompanied by a reduced expression of programmed death 1 (PD-1; Figure S30). These findings suggest that the DREAM treatment elicited systemic immune activation in mice, transforming the TME from an immune-cold to an immune-hot state, which may partially elucidate the mechanism by which DREAM inhibits the distant metastasis of cancer.

**Figure 6:**
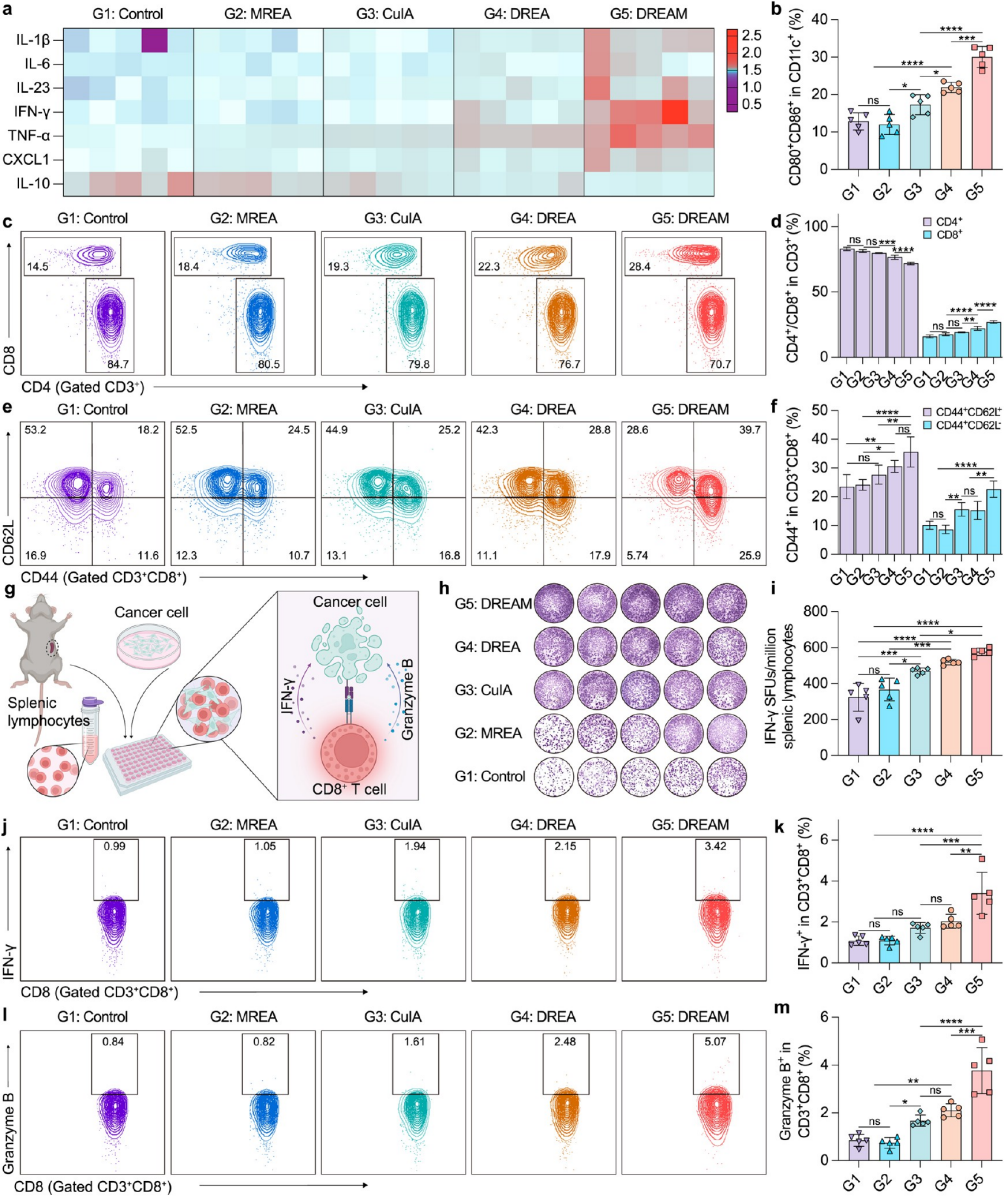
DREAM elicited both innate and adaptive immune responses in SCC7 tumors. (a) Heatmap showing the concentrations of cytokines in the serum of SCC7 tumor-bearing mice with different treatments. The concentration data for cytokines were subjected to normalization through a logarithmic transformation. (b) The percentage of CD80^+^CD86^+^ DCs in tumor-draining lymph nodes of primary tumors. (c, d) Representative FCM plots (c) and the ratios of CD4^+^/CD8^+^ T cells (d) in the spleens of SCC7 tumor-bearing mice. (e, f) Representative FCM plots (e) and the ratios (f) of central memory T cells (T_CM_) and effector memory T cells (T_EM_) in the spleens of SCC7 tumor-bearing mice. (g) Schematic illustration depicting the immune responses of splenic lymphocytes upon *ex vivo* restimulation with SCC7 for 24 h. (h, i) Quantification of IFN-γ spot-forming cells (h) and subsequent statistical analysis (i) were performed on restimulated splenocytes using the enzyme-linked immunosorbent spot (ELISPOT) assay. SFUs, spot-forming units. (j, k) Representative FCM plots (j) and statistical analysis of CD3^+^CD8^+^IFN-γ^+^ T cells (k) in the splenocytes of SCC7 tumor-bearing mice after restimulation with SCC7 cells *ex vivo*. (l, m) Representative FCM plots (l) and statistical analysis of CD3^+^CD8^+^Granzyme B^+^ T cells (m) in the splenocytes of SCC7 tumor-bearing mice after restimulation with SCC7 cells *ex vivo*. Data are shown as the mean ± s.d. (*n* = 5). Statistical significance (b, d, f, i, k, m) was calculated by using one-way ANOVA.

Given the pivotal role of long-term immune memory in preventing tumor recurrence and metastasis, we subsequently evaluated changes in central memory T (T_CM_, defined as CD3^+^CD8^+^CD44^+^CD62L^+^) and effector memory T (T_EM_, defined as CD3^+^CD8^+^CD44^+^CD62L^–^) T cell infiltration within the TME. Tumor-bearing mice treated with DREAM exhibited a significant augmentation in both T_CM_ and T_EM_ cell populations (Figure 6e,f), indicating that DREAM effectively induces immune memory for sustained antitumor protection.^47^ Upon reexposure to the same antigen, a heightened and specific immune response is elicited, characterized by the secretion of IFN-γ and granzyme B, leading to the targeting and subsequent elimination of tumor cells by activated CTLs (Figure 6g). Enzyme-linked immune-spot (ELISPOT) assays revealed a significant increase in IFN-γ-secreting cells in the splenic lymphocytes within the DREAM group following SCC7 cell restimulation *ex vivo* (Figure 6h,i). Consistently, following a second exposure of SCC7 cells, the relative proportion of CD3^+^CD8^+^IFN-γ^+^ T cell population in the spleen cells of the DREAM group (3.40 ± 1.03%) was approximately 1.67 times, 1.99 times, and 3.10 times that of the DREA group (2.04 ± 0.34%), CuIA group (1.71 ± 0.26%), and MREA group (1.10 ± 0.22%), respectively (Figure 6j,k). Furthermore, there was a significant increase in Granzyme B-expressing cells in the DREAM group (Figure 6l,6m), indicating successful establishment of a strong specific CTL immune response by the DREAM nanoplatform in tumor-bearing mice.^48^

### 2.6 DREAM Effectively Inhibited Melanoma Growth and Induced Immune Responses

Reinvigorated by the promising results observed in squamous cell carcinoma, we sought to investigate whether DREAM-based therapy could induce a similar inhibitory effect on the proliferation of other tumor types. The analysis of RNaseq data from the TCGA database revealed that in skin cutaneous melanoma, the expression levels of cuproptosis-related genes (DBT, DLAT, DLD, FDX1, LIPT1, and PDHA1) were significantly higher in tumor tissues compared to normal tissues (Figure 7a), suggesting a potential sensitivity of melanoma to cuproptosis-based therapy. Therefore, a melanoma xenograft model was established by implanting melanoma tumor cells (B16-OVA) into the right flank of C57BL/6 mice on day 0 (Figure 7b). Subsequently, the tumor-bearing mice received *i.t*. injections of PBS, MREA, CuIA, DREA, and DREAM on days 8, 10, and 12, respectively. The tumor volume of the PBS-treated group (Control) exhibited a significant increase, reaching 1152.82 ± 327.19 mm^3^ on the 18th day (Figures 7c, 6d, and S31). Notably, DREAM treatment led to a substantial reduction in tumor size, resulting in a final mean tumor volume of 254.92 mm^3^, while MREA demonstrated a moderate suppression of tumor growth (750.03 mm^3^). Furthermore, the survival of the mice treated with DREAM was significantly prolonged (Figure S32).

**Figure 7:**
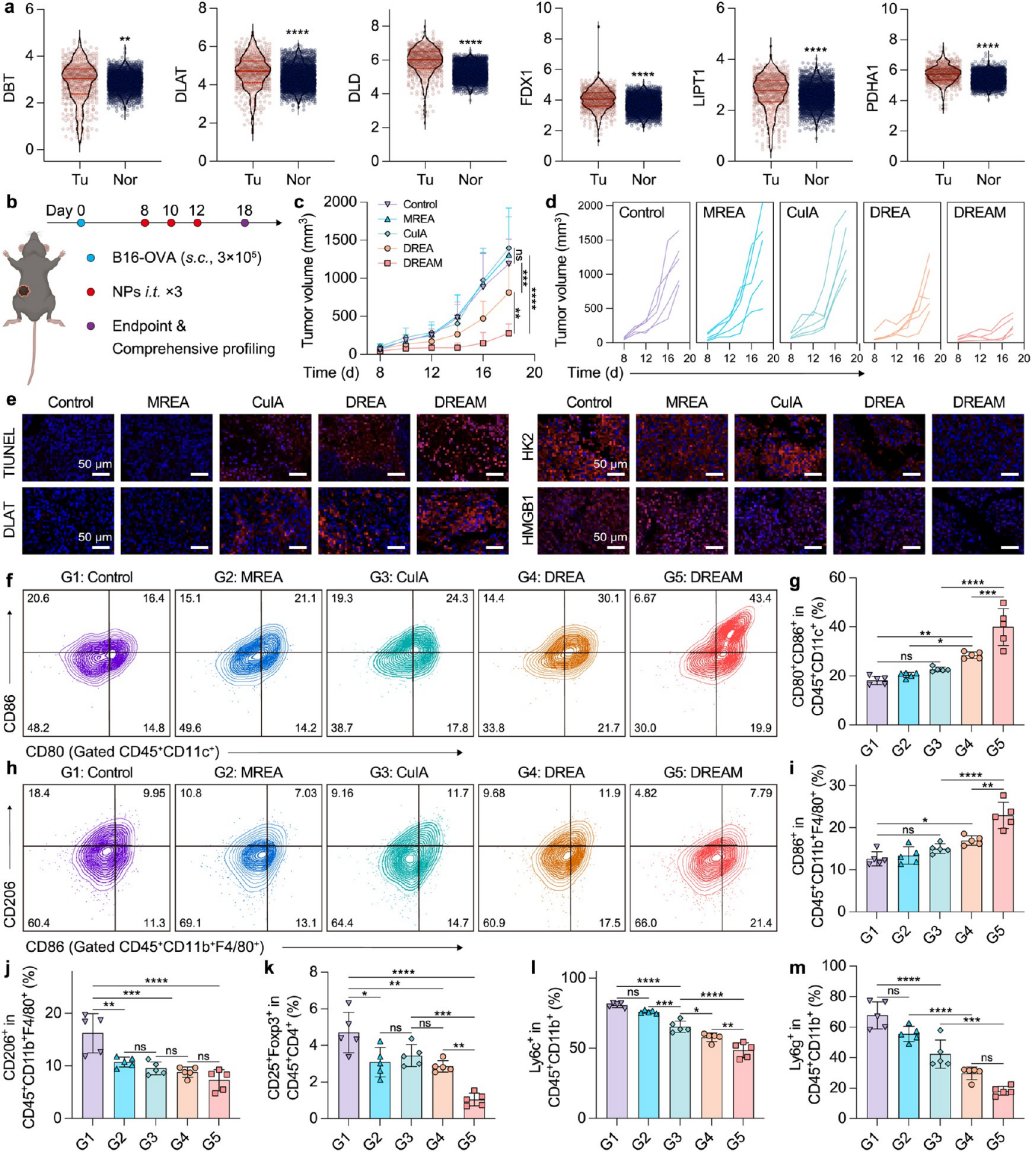
DREAM effectively inhibited B16-OVA tumor growth and induced immune responses *in situ*. (a) The expression distribution of cuproptosis-related genes in melanoma tumor (Tu) tissues and normal (Nor) tissues. Data were obtained from the TCGA data set. (b) Schematic representation of the experimental design for evaluating the antitumor efficacy in melanoma tumors. (c) Average tumor growth curves for primary and distant tumors in B16-OVA tumor-bearing mice with different treatments. (d) Tumor growth kinetics for individual mice. (e) Representative histological sections were stained with TUNEL for apoptosis detection and immunofluorescent staining using HK2, DLAT, and HMGB1 antibodies. Scale bars, 50 μm. (f, g) Representative FCM plots (f) and proportions (g) of CD80^+^CD86^+^ DCs in B16-OVA tumors. (h, i) Representative FCM plots (h) and percentage of M1-type macrophages (i) in tumors. (j–m) Proportions of M2-type macrophages (j), Tregs (k), M-MDSCs (l), and PMN-MDSCs (m) in B16-OVA tumors. Data are shown as the mean ± s.d. (*n* = 5). Statistical significance was determined using the Wilcox test (a), two-way ANOVA (c), and one-way ANOVA (g, i–m).

To further elucidate the underlying antimelanoma mechanisms of the DREAM nanoplatform, tumor tissues were subsequently collected for histological evaluation. The results of H&E and TUNEL staining revealed that DREAM induced the highest level of cell death in B16-OVA tumors (Figure 7e). The immunofluorescence images suggested that DREAM effectively suppressed glycolysis in the TME and induced cupropyl alcohol in tumors. Furthermore, exposure of CRT and release of HMGB1 were significantly elevated in DREAM-treated tumor tissues (Figures S33 and S34), suggesting that DREAM induces ICD in melanoma and holds promise for combating tumor recurrence and metastasis by activating immune responses. Tumors were then harvested, and the proportions of tumor-infiltrating immune cells were assessed to validate that the improved therapeutic effectiveness resulted from the enhancement of systemic immune responses following DREAM treatment. FCM analysis revealed significant increases in the proportions of tumor-infiltrating mature DCs and M1 macrophages (Figure 7f–i), primarily attributed to the potent ability of DREAM to induce cuproptosis and ICD of tumor cells. The formed *in situ* nanovaccines delivered damage-associated molecular patterns (DAMPs) and antigens released by tumor lysis, thereby facilitating efficient activation of antigen-presenting cells and subsequent T cell-mediated immune responses.^31^ The results of immunofluorescence staining indicated a significant increase in the level of infiltration of CD8^+^ T cells and a notable decrease in the level of infiltration of CD4^+^ T cells within tumor tissues treated with the DREAM group, which was accompanied by a reduction in PD-1 expression (Figure S35). Tumor-infiltrating immunosuppressive cells, including M2 macrophages (Figure 7j), Tregs (Figures 7k and S36), M-MDSCs (Figures 7l and S37), and PMN-MDSCs (Figures 7m and S38), were reduced in tumors treated with DREAM. This suggests that DREAM-mediated immune reprogramming reversed the immunosuppressive TME and contributed to further enhancing the long-term efficacy of its combined immunotherapy.^49^

The systemic immune responses were further assessed in tumor-draining lymph nodes and spleens. A significantly higher proportion of mature DCs was confirmed in the tumor-draining lymph nodes of B16-OVA-bearing mice treated with DREAM (Figure 8a,8b). As the tumor specifically expressing ovalbumin (OVA), antigen cross-presentation of SIINKFEL (OVA_257–264_) peptide in B16-OVA tumor-bearing mice promoted the induction of specific CTLs responses by DCs.^50^ Hence, we subsequently investigated the presence of antigen cross-presenting DCs (CD11c^+^SIINFEKL-H-2Kb^+^ DCs) in lymph nodes using FCM analysis, and the results revealed a significantly elevated level of expression of SIINFEKL-H-2Kb in lymph nodes of DREAM-treated mice (Figure 8c,8d), suggesting that DREAM treatment may facilitate the induction of specific immune responses to tumor antigens in mice. Acquired immunity activation can maintain tumor-antigen-specific immune memory, as evidenced by the increased proportion of memory T cells (T_CM_ and T_EM_) observed in the spleen following DREAM treatment (Figure 8e,f). Restimulation with SIINFEKL *ex vivo* significantly raised the levels of CD3^+^CD8^+^IFN-γ^+^ and CD3^+^CD8^+^Granzyme B^+^ T cells in the spleens of DREAM-treated mice (Figure 8g–j), indicating that DREAM triggered tumor-specific immune responses and sustained long-term immune memory.^13^

**Figure 8:**
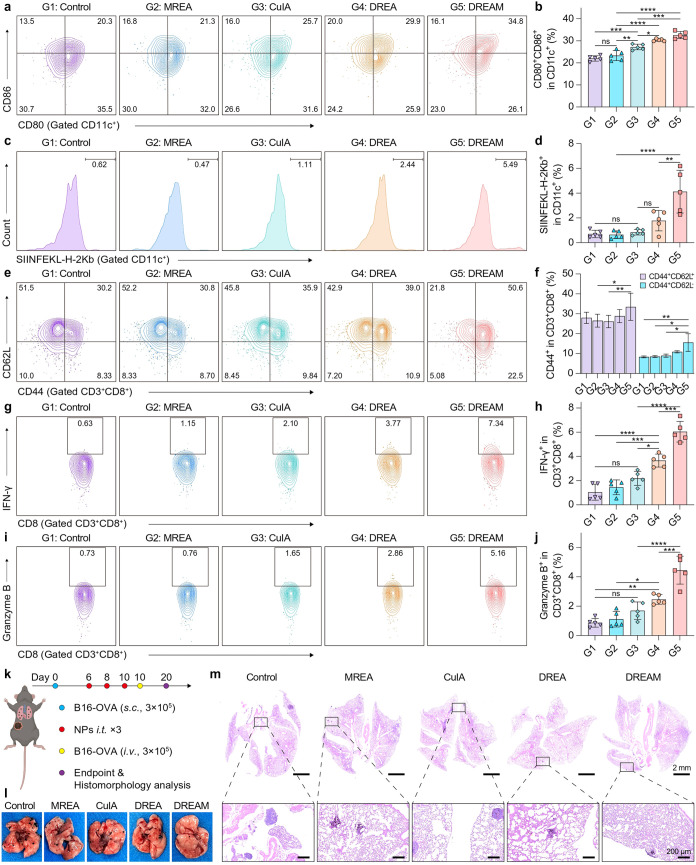
DREAM nanoplatform elicited both innate and adaptive immune responses to inhibit lung metastasis in B16-OVA tumor-bearing mice. (a, b) Representative FCM plots (a) and the ratios of CD80^+^CD86^+^ DCs (b) in tumor-draining lymph nodes. (c, d) Representative FCM histograms (c) and the percentage of SIINFEKL-H-2Kb^+^ DCs (d) in tumor-draining lymph nodes. (e, f) Representative FCM histograms (e) and the ratios of T_CM_ and T_EM_ (f) in spleens of B16-OVA tumor-bearing mice. (g, h) Representative FCM plots (g) and statistical analysis of CD3^+^CD8^+^IFN-γ^+^ T cells (h) in the splenocytes of B16-OVA tumor-bearing mice after restimulation with SIINFEKL *ex vivo*. (i, j) Representative FCM plots (i) and statistical analysis of CD3^+^CD8^+^Granzyme B^+^ T cells (j) in the splenocytes of B16-OVA tumor-bearing mice after restimulation with SIINFEKL *ex vivo*. (k) Schematic representation of the experimental protocol for evaluating the efficacy against lung metastasis. (l, m) Photographs (l) and H&E staining images (m) of metastatic nodules in lungs of mice in various treatment groups. Data are shown as the mean ± s.d. (*n* = 5). Statistical significance (b, d, f, h, j) was calculated by using one-way ANOVA.

To evaluate the potential of the DREAM nanoplatform in activating innate and adaptive immunity to mitigate lung metastasis, B16-OVA tumor-bearing mice were intravenously injected with tumor cells following three treatments with the nanoplatform, thereby establishing lung metastasis models (Figure 8k). A greater number of metastatic nodes were observed in the lung tissues of the control group, whereas the extent of lung metastasis was significantly reduced in the DREAM-treated mice (Figures 8l,m and S39), which suggests that the DREAM nanoplatform is effective in inhibiting tumor metastasis to the lungs.

## 3 Conclusions

In conclusion, we demonstrated that the inhibition of glycolysis may enhance the sensitivity to cuproptosis and augment antitumor immune responses, and targeted modulation of metabolism represents a promising therapeutic strategy for boosting cuproptosis-mediated cancer immunotherapy. To evaluate this hypothesis, a metabolic and immunological dual-remodeling nanoplatform (DREAM) was elaborately constructed, which significantly improved tumor targeting, enhanced intratumoral penetration, and augmented intracellular Cu accumulation, ultimately leading to the induction of cuproptosis in cancer cells. DREAM enhanced the sensitivity of cancer cells to cuproptosis *via* the inhibition of glycolytic signaling, activating the cuproptosis signaling pathway in cancer cells characterized by DLAT aggregation, thereby inducing cancer cell death through proteotoxic stress. Notably, the findings of this study underscored that the DREAM-mediated cuproptosis sensitization strategy could effectively induce ICD, elevate the levels of DAMPs and TAAs in treated tumors, and enhance immune cell infiltration in untreated distant tumors through an *in situ* vaccination effect, thereby significantly inhibiting cancer recurrence and metastasis. Potentially, the proposed strategy of coordinated immunomodulatory enhancement of cuproptosis-mediated immunotherapy in this study may serve to expand therapeutic options for patients with recurrent and metastatic cancer in the future.

## 4 Experimental Section

### 4.1 Bioinformatic Analysis

RNA sequencing data and corresponding clinical information for HNSCC and melanoma tumors were obtained from The Cancer Genome Atlas (TCGA) data set. The expression levels of specific genes in different sample groups were statistically analyzed by using R software (version 4.0.3). The GSVA package in R software was utilized to perform correlation analysis between individual genes and pathway scores, with the parameter method= “ssgsea” specified.^51^ Spearman correlation was subsequently applied to examine the relationship between genes and pathway scores. The immune score was assessed using the EPIC algorithm in the R package immunedeconv,^52^ and the analysis and visualization were conducted utilizing the ggClusterNet package.^53^ The single-cell sequencing analysis data were obtained from the GEO database (GSE172577).^27^ Subsequently, the single-cell data underwent preprocessing and analysis using R software with the packages MAESTRO and Seurat.^54^ The cell clustering was then reorganized by utilizing the tSNE method.

### 4.2 Preparation of the Nanoplatforms

A mixture of 0.16 g of CTAB, 1 mL of aqueous ammonia, and 30 mL of ethanol was dispersed in 75 mL of water and heated to 35 °C in a water bath. After 10 min, 0.25 mL of TEOS was rapidly added and stirred at 35 °C for 30 min. Subsequently, a solution of CuCl_2_ (20 mg/mL) in an amount of 1 mL was added and stirred at 35 °C at a speed of 400 rpm. The products were centrifuged at 8000 rpm, washed with ethanol three times, and then dispersed in a solution of 200 mL of ethanol and 0.1 mL of hydrochloric acid. The extraction process was carried out at 60 °C with stirring at 500 rpm for 3 h, repeated three times. Subsequently, the products were centrifuged and washed with water and ethanol three times to obtain Cu-doped mesoporous organosilica nanoparticles. To load DSF, the Cu-doping mesoporous organosilica nanoparticles (2 mg/mL) were mixed with DSF (8 mg/mL) in 5 mL ethanol at 4 °C for 12 h. The resulting CuIA was then loaded with MREA to obtain DREA for subsequent experiments.

### 4.3 Preparation of Hybrid Membrane-Coated Nanoparticles

Cell membranes were isolated following previously established protocols.^55^ In brief, cells were harvested using cell scrapers and subsequently centrifuged to obtain a pellet. Cells were resuspended in a hypotonic lysis buffer composed of 20 mM Tris-HCl (pH = 7.5), 10 mM KCl, 2 mM MgCl_2_, and EDTA-free mini protease inhibitor tablet (1×). The resulting cell suspension was subsequently transferred to a prechilled Dounce glass homogenizer immersed in an ice bath and subjected to homogenization for 50 cycles, followed by centrifugation at 3200*g* for 5 min. The supernatant was collected, while the pellet was resuspended in hypotonic lysis buffer and underwent an additional 50 cycles of homogenization before being centrifuged once more. The resulting supernatants were combined and further centrifuged at 20,000*g* for 20 min, and the supernatant was further centrifuged at 100,000*g* for 1 h at 4 °C to precipitate cell membrane fragments. M1-type macrophages were generated by incubating RAW264.7 cells with LPS (200 ng/mL) for 24 h. Hybrid membranes, composed of cancer cell membranes (1 mg/mL) and M1-type macrophage membranes (1 mg/mL), were mixed and subjected to physical extrusion through a 400 nm polycarbonate membrane for 13 passes to produce hybrid membrane-derived vesicles. Subsequently, the resulting vesicles were coated onto DREA (5 mg/mL) *via* sonication for 10 min, followed by coextrusion of the vesicles and cores through a 200 nm polycarbonate membrane to form DREAM, which was then stored at 4 °C in PBS for further use.

### 4.4 Tumor Cell Eradication *In Vitro*

SCC7 cells were plated in a 96-well plate with 100 μL of complete medium at a density of 2 × 10^4^ cells per well. Following overnight incubation, the cells were treated with MREA, CuIA, DREA, or DREAM at various concentrations for 24 h. Subsequently, the medium was replaced with fresh medium containing a 10% CCK-8 solution, and the cells were incubated for an additional 1 to 4 h at 37 °C. The optical density of the supernatant was measured at 450 nm using a Synergy HT microplate spectrophotometer to assess cell viability. Besides, SCC7 cells were inoculated with a culture medium supplemented with PBS, MREA (3.3 μg/mL), CuIA (20 μg/mL), DREA, or DREAM (equal amounts of MREA and CuIA). Following a 24 h incubation period, the cells were harvested and subjected to staining using the Annexin V/PI apoptosis assay kit to evaluate cell apoptosis *via* FCM. The BeyoClick EdU Cell Proliferation Kit, conjugated with Alexa Fluor 647, was employed to stain SCC7 cells in order to evaluate their proliferative potential under various treatment conditions, in accordance with the manufacturer’s instructions. For live/dead cell imaging, cells or tumor spheroids were stained using the Calcein/PI cytotoxicity assay kit in accordance with the manufacturer’s protocols, and images were acquired *via* CLSM.

### 4.5 Assessment of Glycolysis, Cuproptosis, and Immunological Cell Death

After the attachment of SCC7 cells (2 × 10^5^ cells/well) in 12-well plates, the cells were treated with fresh medium supplemented with MREA (3.3 μg/mL), CuIA (20 μg/mL), DREA, or DREAM (equal amount of MREA and CuIA) for a period of 24 h. Subsequently, lactate levels in the culture supernatants were measured using a lactate assay kit following the manufacturer’s protocol. The G6P activity assay kit, PA content assay kit, and cell copper (Cu^2+^) colorimetric assay kit were employed to quantify intracellular G6P levels, PA contents, and cellular copper concentrations, respectively. Additionally, the data were standardized following the quantification of the protein concentration in cell lysates using the BCA protein assay kit.

### 4.6 Immunofluorescence Staining Assays

Cells subjected to various treatments were labeled with DiO (5 μM) for 20 min, fixed in 4% PFA for 15 min, permeabilized using 0.125% Triton X-100 for 5 min, and subsequently blocked with 5% BSA for 2 h. Following this, cells were incubated overnight at 4 °C with anti-HK2, anti-PKM2, anti-DLAT, or anti-CRT antibodies and then stained with Alexa Fluor 647-conjugated secondary antibodies for 2 h at room temperature. Nuclei were counterstained with DAPI before observation under CLSM.

### 4.7 Western Blot Assay

The total protein from SCC7 cells subjected to various treatments was extracted using a radioimmunoprecipitation assay (RIPA) lysis buffer supplemented with a protease inhibitor cocktail and quantified by using a BCA protein assay kit. The subsequent protein immunoassay was performed *via* SDS-PAGE electrophoresis, followed by detection through a chemiluminescent imaging system (Tanon, Shanghai, China). The expression levels of HK2, PKM2, ATP7B, LIAS, and HMGB1 were assessed and analyzed, employing β-actin as the internal control.

### 4.8 Biodistribution

SCC7 tumor-bearing mice were randomly allocated to receive intratumoral injections of either DREA-Cy5.5 or DREAM-Cy5.5. Subsequently, the mice were euthanized 24 h postinjection, and major organs along with tumors were harvested for subsequent *ex vivo* imaging utilizing the AniView100 multimodal imaging system (Biolight Biotechnology, Guangzhou, China). The harvested tumor tissues were fixed with 4% PFA and embedded in paraffin. Tissue sections were stained with DAPI and further imaged using CLSM.

### 4.9 Construction of SCC7 Tumor-Bearing Mouse Models and Assessment of Abscopal Therapeutic Effect

Mice were purchased from Vital River Laboratory Animal Technology (Beijing, China). On day 0, primary SCC7 tumors were established by subcutaneously injecting 1 × 10^6^ SCC7 cells into the right flank of C3H/HeN mice (female, 6 week). On day 5, 1 × 10^6^ SCC7 tumor cells were injected subcutaneously into the left flank of primary tumor-bearing mice to establish distant tumors. On day 8, the bilateral tumor-bearing mice were randomly divided into 5 groups (*n* = 5), as follows: Control (G1), MREA (G2), CuIA (G3), DREA (G4), and DREAM (G5). The primary SCC7 tumors received intratumoral injections of PBS, MREA (3.3 μg/mouse), CuIA (20 μg/mouse), DREA, and DREAM (equal amount of MREA and CuIA), followed by reinjections on days 11 and 14. Mice were euthanized on day 24, and the tumors (primary and distant), lymph nodes, spleens, and peripheral blood were harvested to comprehensively assess the immune profiles. The tumor volume was calculated with the formula: tumor volume (mm^3^) = 0.5 × length × width^2^.

### 4.10 Assessment of the Inhibitory Effects on Melanoma Tumor Growth and Lung Metastasis

Each C57BL/6 mouse (female, 6 weeks) was subcutaneously injected in the right flank with a single-cell suspension of 3 × 10^5^ B16-OVA tumor cells in 100 μL of PBS on day 0. Tumor-bearing mice were randomly divided into 5 groups (*n* = 5) and intratumorally injected with PBS, MREA (3.3 μg/mouse), CuIA (20 μg/mouse), DREA, and DREAM (equal amount of MREA and CuIA), on day 8, followed by reinjections on days 10 and 12. All mice were sacrificed on day 18, and the tumors, lymph nodes, and spleens were harvested to assess the immune responses of B16-OVA tumor-bearing mice in each group.

To evaluate lung metastasis, mice were subcutaneously injected with 3 × 10^5^ B16-OVA cells in the right flank on day 0. Following intratumoral treatments administered on days 6, 8, and 10, each tumor-bearing mouse received a tail vein injection of a single-cell suspension containing 3 × 10^5^ B16-OVA cells in 100 μL of PBS on day 10. All mice were euthanized on day 20 to harvest lung tissues for assessment of the inhibitory effects on melanoma lung metastasis.

### 4.11 Statistical Analysis

The data were presented as the mean ± standard deviation (s.d.). The unpaired two-tailed Student’s *t* test was used to assess significant differences between the two groups. Additionally, one- or two-way ANOVA with multiple comparisons was performed as specified. Significant differences were denoted as follows: ns, not significant, *P* > 0.05; *P < 0.05; **P < 0.01; ***P < 0.001; and ****P < 0.0001.

## Supplementary Material



## Data Availability

All data are available in the main text or the Supporting Information.

## Notes

Additional materials and methods; detailed information for the reagents; diameter distribution; XPS analysis; hydrodynamic diameter; polymer dispersity indexes (PDIs); ζ-potentials; the loading efficiency (LE) and encapsulation efficiency (EE) of DSF and MREA; the release kinetics of DSF-Cu and MREA in PBS with different pH values; UV-light excitation images showing the protein composition; quantitative analyses of the MFI in SCC7 cells treated with DREA-Cy5.5 or DREAM-Cy5.5, as detected by FCM analysis; quantitative analyses of apoptotic SCC7 cells; representative FCM histograms and quantitative analyses of SCC7 cells stained with the EdU cell proliferation kit; principal component analysis (PCA) plot; DEG counts for each comparison cluster; heatmap of DEGs; quantitative analyses of the MFI of HK2, PKM2, DLAT, and CRT in SCC7 cells; quantitative analysis of the relative HK2, PKM2, ATP7B, LIAS, and HMGB1 protein expression; representative FCM histograms and quantitative analyses of SCC7 cells expressing CRT on cell membrane; released HMGB1 in the cell culture supernatant; quantification of the fluorescence intensity in the tumors; tumor photographs of primary and distant SCC7 tumors; survival curves of SCC7-bearing mice; mouse weight of the SCC7 tumor-bearing mice; H&E staining of major organs; representative immunofluorescent images showing SCC7 tumor sections were stained with the CRT antibodies; quantitative analyses of the MFI of CRT in tumor tissues; percentage of TUNEL^+^ cells in tumor tissues harvested from SCC7 tumor-bearing mice; quantitative analyses of the MFI of HK2, DLAT, and HMGB1 in tumor tissues harvested from SCC7 tumor-bearing mice; representative FCM plots of Tregs in both primary and distant SCC7 tumors; representative FCM plots of M-MDSCs in both primary and distant SCC7 tumors; representative FCM plots of PMN-MDSCs in both primary and distant SCC7 tumors; the concentrations of cytokines detected by cytometric bead array in the serum; representative FCM plots and the percentage of CD80^+^CD86^+^ DCs in tumor-draining lymph nodes; representative immunofluorescent images showing SCC7 tumor sections were stained with the CD4, CD8, and PD-1 antibodies; percentage of CD4^+^, CD8^+^, and PD-1^+^ T cells in tumor tissues harvested from SCC7 tumor-bearing mice; tumor photographs of B16-OVA tumors; survival curves of B16-OVA-bearing mice; representative immunofluorescent images showing B16-OVA tumor sections stained with the CRT antibodies; quantitative analyses of the MFI of CRT in tumor tissues harvested from B16-OVA tumor-bearing mice; percentage of TUNEL^+^ cells in tumor tissues harvested from B16-OVA tumor-bearing mice; quantitative analyses of the MFI of HK2, DLAT, and HMGB1 in tumor tissues harvested from B16-OVA tumor-bearing mice; representative immunofluorescent images showing B16-OVA tumor sections were stained with the CD4, CD8, and PD-1 antibodies; percentage of CD4^+^, CD8^+^, and PD-1^+^ T cells in tumor tissues harvested from B16-OVA tumor-bearing mice; representative FCM plots of Tregs in B16-OVA tumors; representative FCM plots of M-MDSCs in B16-OVA tumors; representative FCM plots of PMN-MDSCs in B16-OVA tumors; and numbers of lung metastatic nodules (PDF)

## Abbreviations

TME: tumor microenvironment
DREAM: dual-remodeling cuproptosis-inducing agent with hybrid cell membrane coating
TCA: tricarboxylic acid
Fe–S: iron–sulfur
DAMPs: damage-associated molecular patterns
TAAs: tumor-associated antigens
DCs: dendritic cells
CTLs: cytotoxic T lymphocytes
CNPs: cell membrane-coated nanoparticles
TAMs: tumor-associated macrophages
DSF: disulfiram
3-BA: 3-bromopyruvic acid
HNSCC: head and neck squamous cell carcinoma
TCGA: The Cancer Genome Atlas
LIPT1: lipoyltransferase 1
DLST: dihydrolipoamide S-succinyltransferase
GCSH: glycine cleavage system protein H
DLAT: dihydrolipoamide S-acetyltransferase
FDX1: ferredoxin 1
LIAS: lipoic acid synthetase
NK: natural killer
CuIA: cuproptosis-inducing agent
LPS: lipopolysaccharide
CLSM: confocal laser scanning microscopy
FCM: flow cytometry
DEGs: differentially expressed genes
GO: gene ontology
IFN-β: interferon-β
ICD: immunogenic cell death
NF-κB: I-kappaB kinase/nuclear factor kappa-B
TNF: tumor necrosis factor
HK2: hexokinase 2
PKM2: pyruvate kinase isozyme type M2
ER: endoplasmic reticulum
CRT: calreticulin
HMGB1: high mobility group protein 1
Tregs: regulatory T cells
MDSCs: myeloid-derived suppressor cells
ELISPOT: enzyme-linked immune-spot
OVA: ovalbumin
