# Supplementary material for: Hybrid Cell Membrane-Functionalized Nanoagents Synergistically Enhance Cuproptosis-Mediated Immunotherapy by Dual Modulation of Glycolytic Metabolism and Tumor Microenvironments
Source: ACS Nano. 2025 Aug 4;19(31):28913–32. doi: 10.1021/acsnano.5c10671 (PMC12356196; doi:10.1021/acsnano.5c10671)
Supplement: Supplementary file 1 [file nn5c10671_si_001.pdf]

Supporting Information

Hybrid Cell Membrane-Functionalized Nanoagents Synergistically Enhance Cuproptosis-Mediated Immunotherapy by Dual Modulation of Glycolytic Metabolism and Tumor Microenvironments

Qiang Li[†1], Meng Dang[†1], Ao He[†1], Xiaoye Li[1], Meng Ding[1], Zhuo Dai[1], Yu Zhang[1], Weijun Xiu[2], Siyu Wang[3], Zhusheng Huang[4], Yongbin Mou[1], Lianhui Wang[4*], Heng Dong[1*]*

[1]Nanjing Stomatological Hospital, Affiliated Hospital of Medical School, Institute of Stomatology, Nanjing University, 30 Zhongyang Road, Nanjing, Jiangsu 210008, China.

[2]Institute for Health Innovation & Technology, Biomedical Engineering Department, National University of Singapore, 21 Lower Kent Ridge Road, Singapore 119276, Singapore.

[3]Marc and Jennifer Lipschultz Precision Immunology Institute, Icahn School of Medicine at Mount Sinai, 1 Gustave L Levy Pl, New York, NY 10029, USA.

[4]State Key Laboratory for Organic Electronics and Information Displays & Jiangsu Key Laboratory for Biosensors, Institute of Advanced Materials (IAM), Jiangsu National Synergistic Innovation Center for Advanced Materials (SICAM), Nanjing University of Posts and Telecommunications, 9 Wenyuan Road, Nanjing, Jiangsu 210023, China.

[†]These authors contributed equally: Qiang Li, Meng Dang, Ao He

*Corresponding author. yongbinmou@nju.edu.cn (Y. Mou), iamlhwang@njupt.edu.cn (L. Wang), dongheng90@smail.nju.edu.cn (H. Dong)

CONTENTS

Table S1. Detailed information for the reagents.

Figure S1. Diameter distribution of the DREA.

Figure S2. X-ray photoelectron spectroscopy (XPS) analysis of DREA.

Figure S3. Hydrodynamic diameter of CuMON, CuIA, DREA, and DREAM.

Figure S4. Polymer dispersity indexes (PDIs) of CuMON, CuIA, DREA, and DREAM.

Figure S5. Zeta potentials of CuMON, CuIA, DREA, and DREAM.

Figure S6. a,b) The loading efficiency (LE) and encapsulation efficiency (EE) of DSF **(a)** and MREA **(b)** within the DREAM nanoplatform. **c,d)** The release kinetics of DSF-Cu **(c)** and MREA **(d)** in PBS with different pH values.

Figure S7. UV-light excitation images showing the protein composition of M1-macrophage membrane (mM), cancer cell membrane (mC), DREA, and DREAM on gels stained with One-Step Lumitein™ UV protein gel dye after SDS-PAGE electrophoresis.

Figure S8. Quantitative analyses of the mean fluorescence intensity (MFI) in SCC7 cells treated with DREA-Cy5.5 or DREAM-Cy5.5 for 1, 3, or 6 hours, as detected by FCM analysis.

Figure S9. Quantitative analyses of apoptotic SCC7 cells after incubation with PBS (Control), MREA, CuIA, DREA, or DREAM for 24 h, as determined by FCM analysis using an Annexin V-FITC/PI apoptosis assay kit.

Figure S10. Representative FCM histograms (**a**) and quantitative analyses (**b**) of SCC7 cells stained with the 5-ethynyl-2'-deoxyuridine (EdU) cell proliferation kit.

Figure S11. Principal component analysis (PCA) plot showed SCC7 cells with different treatments separated by their top three principal components.

Figure S12. Differentially expressed gene (DEG) counts for each comparison cluster.

Figure S13. Heatmap of DEGs among SCC7 cells treated with PBS (Control), MREA, CuIA, DREA, and DREAM.

Figure S14. Quantitative analyses of the MFI of HK2, PKM2, DLAT, and CRT in SCC7 cells under different treatment conditions, as quantified using ImageJ based on CLSM images.

Figure S15. Quantitative analysis of the relative HK2, PKM2, ATP7B, LIAS, and HMGB1 protein expression in SCC7 cells treated with PBS (Control), MREA, CuIA, DREA, or DREAM for 24 hours, as quantified using ImageJ based on western blot data.

Figure S16. Representative FCM histograms (**a**) and quantitative analyses (**b**) of SCC7 cells expressing CRT on cell membrane.

Figure S17. Released HMGB1 in the cell culture supernatant in SCC7 cells treated with PBS (Control), MREA, CuIA, DREA, or DREAM for 24 h, as quantified by enzyme-linked immunosorbent assay (ELISA).

Figure S18. Quantification of the fluorescence intensity in the tumors from mice 24 hours after i.t. administration of DREA-Cy5.5 or DREAM-Cy5.5.

Figure S19. Tumor photographs of primary (a) and distant (b) SCC7 tumors with the indicated treatments (n = 5).

Figure S20. Survival curves of SCC7-bearing mice following the indicated treatments.

Figure S21. Mouse weight of the SCC7 tumor-bearing mice with the indicated treatments.

Figure S22. Hematoxylin and eosin (H&E) staining of major organs (heart, liver, spleen, lung, and kidney) from SCC7 tumor-bearing mice after treatment as indicated.

Figure S23. **a)** Representative immunofluorescent images showing SCC7 tumor sections were stained with the CRT (red) antibodies. Scale bars, 50 μm. **b)** Quantitative analyses of the MFI of CRT in tumor tissues harvested from SCC7 tumor-bearing mice.

Figure S24. **a)** Percentage of $TUNEL^+$ cells in tumor tissues harvested from SCC7 tumor-bearing mice. **b-d)** Quantitative analyses of the MFI of HK2(**b**), DLAT(**c**), and HMGB1(**d**) in tumor tissues harvested from SCC7 tumor-bearing mice.

Figure S25. Representative FCM plots of Tregs ($CD45^+CD4^+CD25^+Foxp3^+$) in both primary and distant SCC7 tumors.

Figure S26. Representative FCM plots of M-MDSCs ($CD45^+CD11b^+Ly6c^+$) in both primary and distant SCC7 tumors.

Figure S27. Representative FCM plots of PMN-MDSCs ($CD45^+CD11b^+Ly6g^+$) in both primary and distant SCC7 tumors.

Figure S28. The concentrations of cytokines detected by cytometric bead array in the serum of tumor-bearing mice with different treatments.

Figure S29. Representative FCM plots and the percentage of $CD80^+CD86^+$ DCs in tumor-draining lymph nodes ($CD11c^+CD80^+CD86^+$) of primary SCC7 tumors.

Figure S30. a) Representative immunofluorescent images showing SCC7 tumor sections were stained with the CD4 (green), CD8 (red), and PD-1 (red) antibodies. Scale bars, 50 μm. **b-d)** Percentage of $CD4^+$ (**b**), $CD8^+$ (**c**), and PD-1^+ (**d**) T cells in tumor tissues harvested from SCC7 tumor-bearing mice.

Figure S31. Tumor photographs of B16-OVA tumors with the indicated treatments.

Figure S32. Survival curves of B16-OVA-bearing mice following the indicated treatments.

Figure S33. a) Representative immunofluorescent images showing B16-OVA tumor sections were stained with the CRT (red) antibodies. Scale bars, 50 μm. **b)** Quantitative analyses of the MFI of CRT in tumor tissues harvested from B16-OVA tumor-bearing mice.

Figure S34. a) Percentage of $TUNEL^+$ cells in tumor tissues harvested from B16-OVA tumor-bearing mice. **b-d)** Quantitative analyses of the MFI of HK2(**b**), DLAT(**c**), and HMGB1(**d**) in tumor tissues harvested from B16-OVA tumor-bearing mice.

Figure S35. a) Representative immunofluorescent images showing B16-OVA tumor sections were stained with the CD4 (green), CD8 (red), and PD-1 (red) antibodies. Scale bars, 50 μm. **b-d)** Percentage of $CD4^+$ (**b**), $CD8^+$ (**c**), and $PD\text{-}1^+$ (**d**) T cells in tumor tissues harvested from B16-OVA tumor-bearing mice.

Figure S36. Representative FCM plots of Tregs ($CD45^+CD4^+CD25^+Foxp3^+$) in B16-OVA tumors.

Figure S37. Representative FCM plots of M-MDSCs ($CD45^+CD11b^+Ly6c^+$) in B16-OVA tumors.

Figure S38. Representative FCM plots of PMN-MDSCs ($CD45^+CD11b^+Ly6g^+$) in B16-OVA tumors

Figure S39. Numbers of lung metastatic nodules.

Supplementary Experimental Section

Reagents

Detailed information on the chemicals, critical commercial assays, and antibodies is provided in **Table S1**.

Table S1. Detailed information for the reagents.

REAGENT or RESOURCE	SOURCE	IDENTIFIER
**Chemicals, Peptides, and Recombinant Proteins**		
Anhydrous ethanol	Sinopharm Chemical Reagent	L04413401
Cetyltrimethylammonium bromide (CTAB)	Sinopharm Chemical Reagent	30037416
Concentrated ammonia aqueous solution ($NH_3 \cdot H_2O$, 25~28 wt%)	Sinopharm Chemical Reagent	10002108
Tetraethyl orthosilicate (TEOS)	Sinopharm Chemical Reagent	80124118
Copric chloride dihydrate ($CuCl_2$)	Sigma–Aldrich	451665
Bis[3-(triethoxysilyl)propyl] tetrasulfide (TESPTS, 90 wt%)	Sigma–Aldrich	15200
Disulfiram (DSF)	MedChemExpress	HY-B0240
3-Bromopyruvic acid (BA)	Selleck	S5426
Collagenase I	BioFroxx	1904MG100
Collagenase IV	BioFroxx	2091MG100
Hyaluronidase	BioFroxx	1141GR001
DNase I	BioFroxx	1121MG100
DAPI-containing anti-fluorescence quencher	Beyotime	P0131
TRIzol reagent	Invitrogen	15596-026
**Antibodies**		
Hexokinase 2 polyclonal antibody	Proteintech	22029-1-AP
DLAT mouse mAb	Cell Signaling Technology	12362
PKM2-specific polyclonal antibody	Proteintech	15822-1-AP
FDX1 polyclonal antibody	Proteintech	12592-1-AP
ATP7B-specific polyclonal antibody	Proteintech	19786-1-AP
LIAS polyclonal antibody	Proteintech	11577-1-AP
Calreticulin recombinant rabbit monoclonal antibody	Invitrogen	MA5-32131
HMGB1 recombinant rabbit monoclonal antibody	Invitrogen	MA5-31967
β-Actin Antibody	Abmart	P30002L
Goat anti-rabbit mouse IgG-HRP	Abmart	M21003S
Goat anti-mouse IgG-HRP	Abmart	M21001S

Anti-CD80-PE	eBioscience	12-0801-82
Anti-CD86-APC	eBioscience	17-0862-82
Anti-CD86-PE	eBioscience	12-0862-82
Anti-CD206-APC	eBioscience	17-2061-82
Anti-CD3-APC	eBioscience	17-0031-82
Anti-CD4-FITC	eBioscience	11-0041-82
Anti-CD8-PercpCy5.5	eBioscience	45-0081-82
Anti-CD44-FITC	eBioscience	11-0441-82
Anti-CD62L-PercpCy5.5	eBioscience	45-0621-82
Anti-IFN-γ-PE	eBioscience	12-7311-82
Anti-CD45-PE-Cy7	eBioscience	25-0451-82
Anti-CD11c-AF700	eBioscience	56-0114-82
Anti-CD25-APC	eBioscience	17-0251-82
Anti-Foxp3-PE	eBioscience	12-5773-82
Anti-Granzyme B-PE	eBioscience	12-8898-82
Anti-Ly6c-APC	eBioscience	17-5932-82
Anti-Ly6g-PE	eBioscience	12-9668-82
Anti-SIINKFEL-H-2Kb-PE	BioLegend	141604
Purified anti-mouse CD16/32 antibody	BioLegend	101302
**Critical commercial assays**		
Cell counting kit-8 (CCK-8)	Dojindo	CK04
BCA protein assay kit	Thermo Fisher	23227
IFN-γ enzyme-linked immunosorbent spot (ELISPOT) kit	Abcam	AB64029
BD Cytofix/Cytoperm™ and fixation/permeabilization kit	BD Biosciences	554714
Calcein/PI cell viability/cytotoxicity assay kit	Beyotime	C2015M
Annexin V-FITC/PI apoptosis kit	MultiSciences	AP101
BeyoClick™ EdU cell proliferation kit with Alexa Fluor 647	Beyotime	C0081S
Lactic acid (LA) content assay kit	Solarbio	BC2235
Pyruvate (PA) content assay kit	Solarbio	BC2205
Glucose-6-phosphatase (G6P) activity assay kit	Solarbio	BC3325
Cell copper (Cu^{2+}) colorimetric assay kit	Elabscience	E-BC-K775-M
RayPlex Mouse Inflammation Bead Array 1	RayBiotech	FAM-IFN-1
One step TUNEL apoptosis assay kit	Beyotime	C1090

Short hairpin RNA (shRNA)-mediated knockdown of HK2

Lentiviral pLKO.1-shRNA vectors targeting mouse HK2 were constructed. For lentiviral packaging, a PEI-based co-transfection system was employed with the plasmid ratio of pMD2.G: psPAX2: target plasmid set at 1: 3: 4. Specifically, pLKO.1-SCR and pLKO.1-HK2-sh plasmids

were transfected into HEK-293T cells using this system. The shRNA sequences were designed and synthesized by GeneScript (Nanjing, China), with the TRC catalog number: TRCN0000012545. At 48 hours post-transfection, the culture supernatants were collected and filtered through a 0.45 μm filter. When SCC7 cells reached approximately 50% confluency in a 6-well plate, they were infected with a mixture of 1 mL of lentiviral supernatant and 1 mL of fresh culture medium. Following a 24-hour incubation period, the SCC7 cells were selected using puromycin (6 μg/mL) for 2 days to ensure successful infection.

Characterization

Transmission electron microscopy (TEM, HT7700 microscope, Tokyo, Japan) was conducted at an accelerating voltage of 100 kV, while scanning electron microscopy (SEM, S4800 microscope, Tokyo, Japan) was performed at an accelerating voltage of 3 kV to characterize the morphology of the nanoparticles. UV-vis absorption spectra were recorded using a PerkinElmer Lambda 365 spectrometer (PerkinElmer, Billerica, MA, USA). The zeta potentials and hydrodynamic sizes of the nanoparticles were determined with a Brookhaven ZetaPALS analyzer (Brookhaven Instruments, Holtsville, NY, USA). X-ray photoelectron spectroscopy (XPS) analysis was carried out using a PHI 5000 VersaProbe (ULVAC-PHI, Chigasaki, Japan).

Cellular internalization

To explore the intracellular colocalization of hybrid cell membranes within cancer cells, DREA was conjugated with Cy5.5 to generate DREA-Cy5.5, which was subsequently coated with DiI-labeled M1-macrophage membrane (mM-DiI) and/or DiO-labeled cancer cell membrane (mC-DiO). In brief, DREA-Cy5.5 was synthesized by stirring 1 mg of DREA with 0.1 mg of Cy5.5 for 12 hours at 4 °C in the dark, followed by three washes with H_2O. DiI and DiO were employed to label LPS-stimulated RAW264.7 and SCC7 cells, respectively. Fluorescently labeled cell

membranes and nanoparticles were prepared following the aforementioned protocols. Subsequently, SCC7 cells (1×10^5 per dish) were cultured in confocal dishes and treated with DREA-Cy5.5, DREA-Cy5.5@mM-DiI, DREA-Cy5.5@mC-DiO, or DREA-Cy5.5@mM-DiI&mC-DiO for 3 hours. The nucleus was stained using DAPI, and imaging was conducted utilizing a Nikon A1 confocal laser scanning microscopy (CLSM; Nikon, Tokyo, Japan).

To further investigate the benefits of hybrid membrane-coated nanoparticles in enhancing tumor cell uptake and tissue penetration, we subsequently synthesized DREA-Cy5.5 as well as DREA-Cy5.5 encapsulated within fusion membranes (DREAM-Cy5.5). DREA-Cy5.5 or DREAM-Cy5.5 (equal amount of DREA-Cy.5.5, 10 μg/mL) was added to SCC7 medium, and the cells were further co-cultured for 1, 3, and 6 hours. The mean fluorescence intensity (MFI) of Cy5.5 in the cells was further detected by flow cytometry (FCM). Alternatively, SCC7 cells were plated in confocal dishes at a density of 2×10^5 and incubated overnight to facilitate cell adhesion. DREA-Cy5.5 or DREAM-Cy5.5 was introduced to the SCC7 cells and allowed to incubate for durations of 1, 3, and 6 hours. Subsequently, the cells were fixed with 4% paraformaldehyde (PFA) for 15 minutes at room temperature, washed three times with PBS, permeabilized using 0.125% Triton X-100 in PBS, and then washed again with PBS. The cells were stained with fluorescein isothiocyanate (FITC)-labeled phalloidin (50 μg/mL) and incubated for 1 hour at room temperature in the dark. Finally, the cell nuclei were stained using DAPI before imaging on the CLSM.

To culture 3D tumor spheroids, cells were initially grown in DMEM supplemented with 10% FBS, 1% penicillin-streptomycin (P/S), and 2.5% Matrigel Matrix within a 96-well plate pre-coated with 3 wt% agarose. The culture medium was refreshed on days 3, 5, and 7, after which the SCC7 tumor spheroids were utilized for subsequent studies on day 7. DREA-Cy5.5 or DREAM-Cy5.5 (equal amount of DREA-Cy.5.5, 10 μg/mL) was introduced into the medium of SCC7 tumor spheroids, followed by co-culturing for durations of 1, 3, and 6 hours. CLSM was employed to capture the fluorescence signal of Cy5.5 at varying depths within the 3D tumor spheroids.

RNA-seq analysis

RNA was extracted from cells by using the TRIzol Reagent according to the manufacturer's instructions. The initial step in establishing the sequencing library involved utilizing the NEBNext® Ultra™ II RNA Library Prep Kit for Illumina® (New England Biolabs). The purified products were quantified using the Agilent high-sensitivity DNA assay on a Bioanalyzer 2100 system. Subsequently, sequencing of the library was performed using an Illumina NovaSeq 6000 platform. Raw data quality control was conducted with FastQC.[1] The sequenced reads were aligned to the mouse reference genome using HISAT2,[2] and messenger RNA expression levels were quantified based on the aligned reads with HTSeq.[3] Differential expression analysis of count data was carried out using DESeq2,[4] with criteria for identifying differentially expressed genes set at $|\log_2\text{Fold-Change}| > 1$ and significant P-value < 0.05. Gene Ontology (GO) enrichment analyses were performed using DAVID.[5]

Histomorphology analysis of tumors

Tumors were prepared into paraffin sections and then stained for TUNEL assays. For immunofluorescence staining, tumor sections were deparaffinized, subjected to antigen retrieval, blocked with BSA, incubated with primary antibodies (anti-HK2 antibody, anti-DLAT antibody, or anti-HMGB1 antibody), and added the corresponding fluorescent dye-conjugated secondary antibodies following the manufacturer's instructions. Nuclei were further stained with DAPI.

Assessment of immune responses *in vivo*

SCC7 tumors were meticulously dissected into small fragments and subsequently digested with enzymes at 37 ˚C for 30 minutes in RPMI 1640 supplemented with 1% penicillin-streptomycin,

1.5 mg/mL collagenase I, 1.5 mg/mL collagenase IV, 1.5 mg/mL hyaluronidase, and 0.2 mg/mL DNase I. The enzymatically hydrolyzed SCC7 tumor tissues were ground and filtered through 70 μm cell strainers to obtain single-cell suspensions. For B16-OVA tumors, single-cell suspensions were obtained by grinding and filtering the tumor tissues directly through 70 μm cell strainers without enzymatic hydrolysis. The isolated cells were initially blocked using anti-CD16/CD32 antibodies to prevent non-specific binding to Fc receptors, followed by staining with the specified FCM antibodies.

Macrophages were stained with anti-CD45-PE-Cy7, anti-CD11b-FITC, anti-F4/80-PercpCy5.5, anti-CD86-PE, anti-CD206-APC; DCs were marked by anti-CD45-PE-Cy7, anti-CD11c-Alexa Fluor 700, anti-CD80-PE, and anti-CD86-APC; Tregs: anti-CD45-PE-Cy7, anti-CD4-FITC, anti-CD25-APC, anti-Foxp3-PE (intracellular staining); MDSCs: anti-CD45-PE-Cy7, anti-CD11b-FITC, anti-Ly6g-PE, anti-Ly6c-APC. Splenic lymphocytes were stained with anti-CD3-APC, anti-CD8-PE, and anti-CD4-FITC to identify the subtypes of T cells. Moreover, anti-CD3-APC, anti-CD8-PE, anti-CD44-FITC, and anti-CD62L-PercpCy5.5 were used to assess the percentage of memory T cells.

The RayPlex Mouse Inflammation Bead Array 1 was employed to evaluate inflammatory cytokine levels in the serum of tumor-bearing mice, following the protocol provided by the manufacturer. To further evaluate the activation of specific immune responses mediated by CTLs, lymphocytes from the spleen were seeded at a density of 1×10^6 cells per well in a 96-well plate and cocultured with 2×10^5 SCC7 cells or SIINFEKL ($OVA_{257-264}$,1 μg/mL) for 12 h. Following another 6 h of co-culture in RPMI-1640 supplemented with 10% FBS, 1% P/S, brefeldin A/monensin mixture, and PMA/ionomycin mixture at 37 °C, intracellular cytokine staining was performed using FCM to detect intracellular IFN-γ and Granzyme B levels. Specifically, the restimulated splenocytes were stained with anti-CD3-APC and anti-CD8-PercpCy5.5 antibodies for surface staining, fixed and permeabilized using a BD Cytofix/Cytoperm kit, and intracellularly

stained with an anti-IFN-γ-PE or anti-Granzyme B-PE antibodies in BD Perm/Wash™ buffer. IFN-γ released from splenic T cells *ex vivo* co-culture in the SCC7 cellular environment was also measured using an IFN-γ ELISPOT kit.

Supplementary Figures

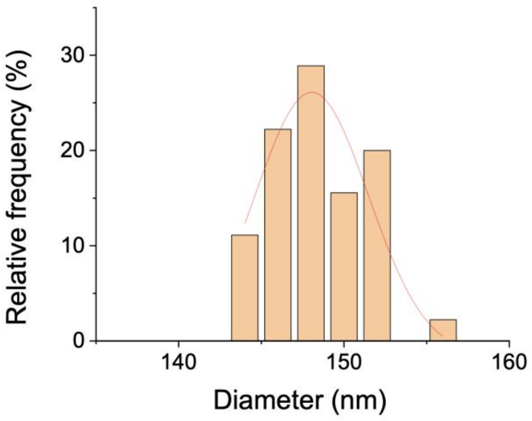

Figure S1. Diameter distribution of the DREA.

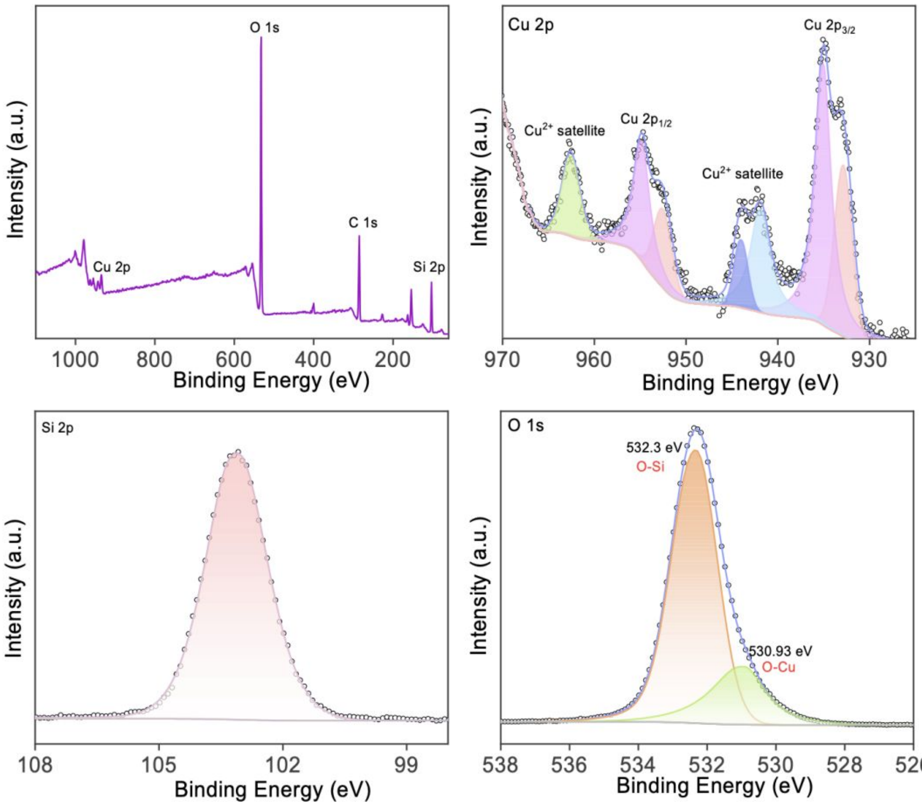

Figure S2. X-ray photoelectron spectroscopy (XPS) analysis of DREA.

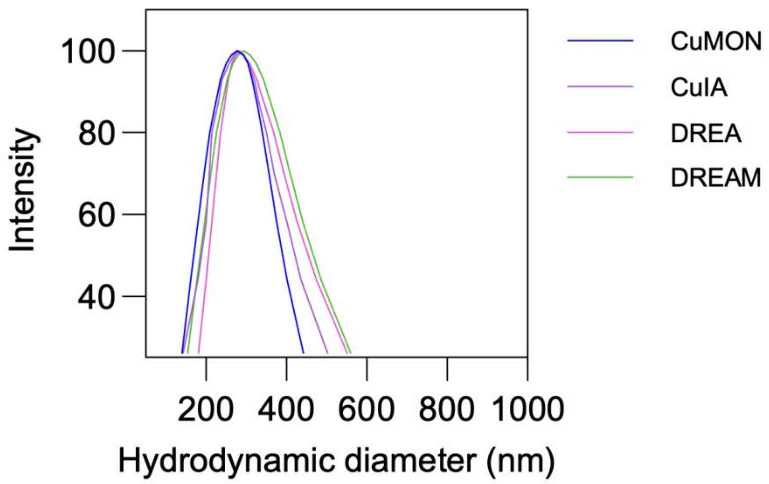

Figure S3. Hydrodynamic diameter of CuMON, CuIA, DREA, and DREAM.

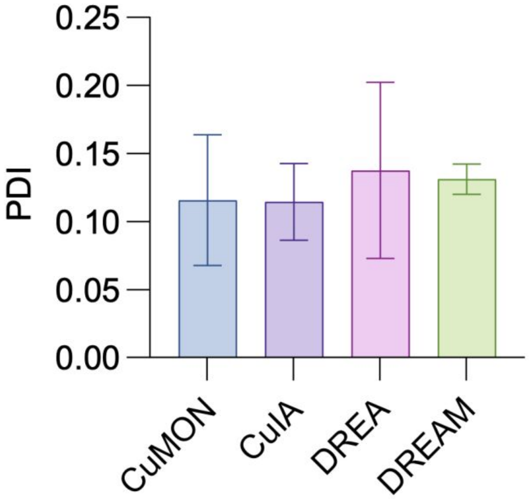

Figure S4. Polymer dispersity indexes (PDIs) of CuMON, CuIA, DREA, and DREAM.

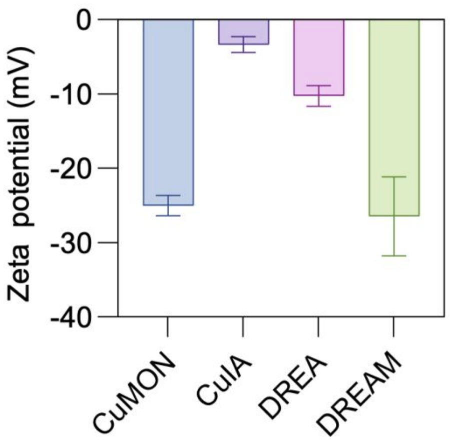

Figure S5. Zeta potentials of CuMON, CuIA, DREA, and DREAM.

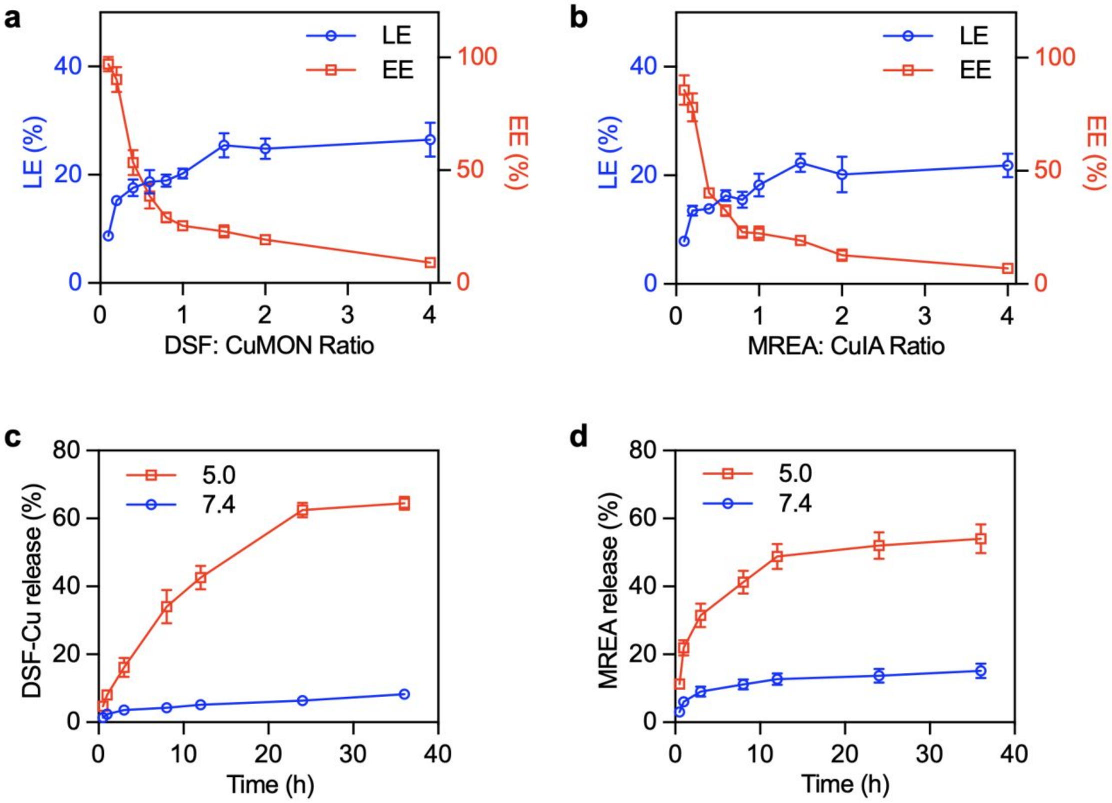

Figure S6. a,b) The loading efficiency (LE) and encapsulation efficiency (EE) of DSF **(a)** and MREA **(b)** within the DREAM nanoplatform. **c,d)** The release kinetics of DSF-Cu **(c)** and MREA **(d)** in PBS with different pH values.

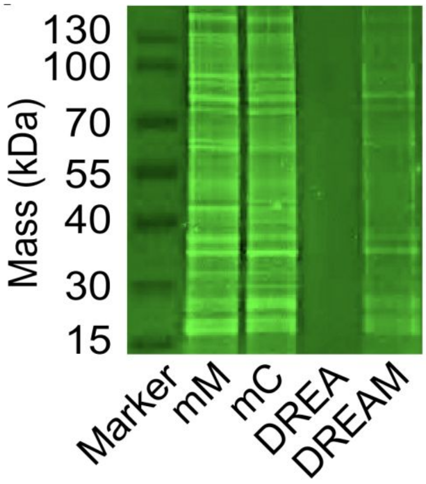

Figure S7. UV-light excitation images showing the protein composition of M1-macrophage membrane (mM), cancer cell membrane (mC), DREA, and DREAM on gels stained with One-Step Lumitein™ UV protein gel dye after SDS-PAGE electrophoresis.

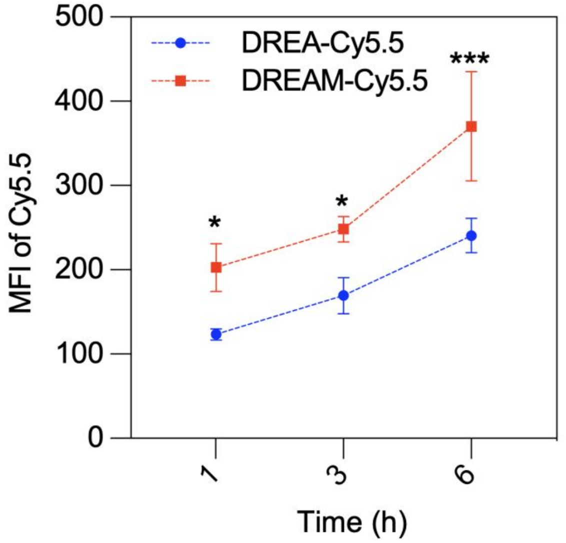

Figure S8. Quantitative analyses of the mean fluorescence intensity (MFI) in SCC7 cells treated with DREA-Cy5.5 or DREAM-Cy5.5 for 1, 3, or 6 hours, as detected by FCM analysis.

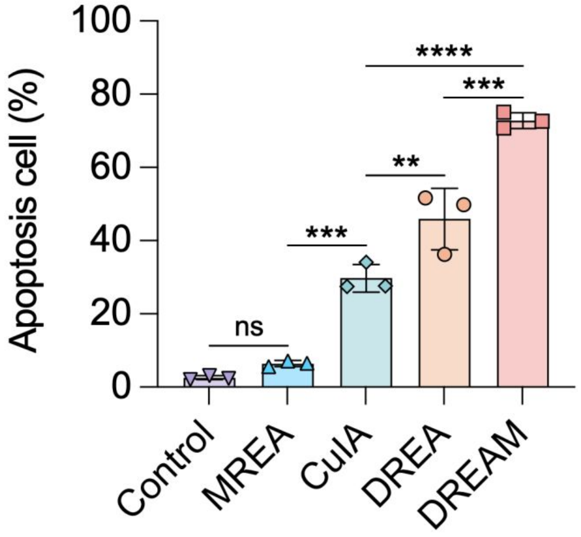

Figure S9. Quantitative analyses of apoptotic SCC7 cells after incubation with PBS (Control), MREA, CuIA, DREA, or DREAM for 24 h, as determined by FCM analysis using an Annexin V-FITC/PI apoptosis assay kit.

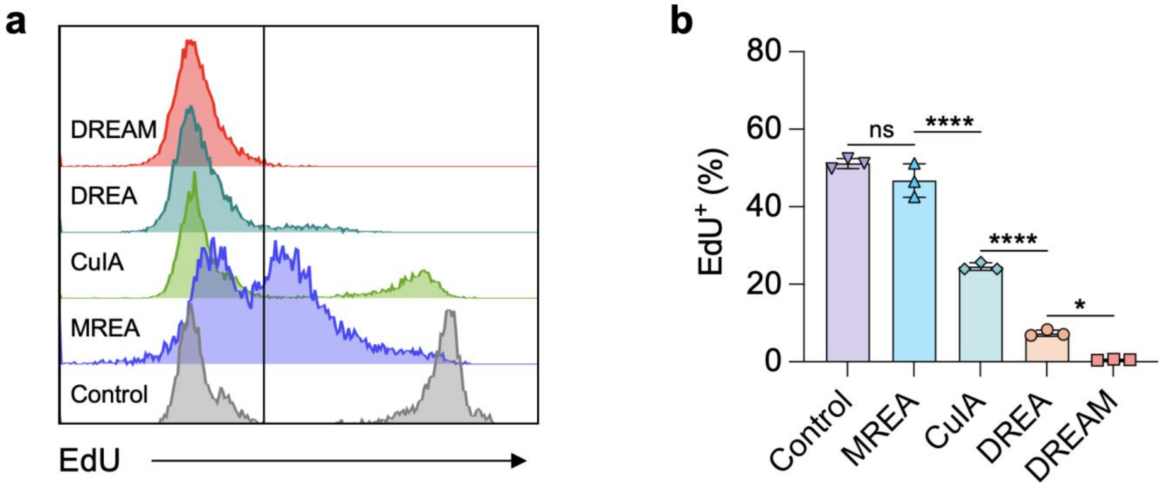

Figure S10. Representative FCM histograms (**a**) and quantitative analyses (**b**) of SCC7 cells stained with the 5-ethynyl-2'-deoxyuridine (EdU) cell proliferation kit.

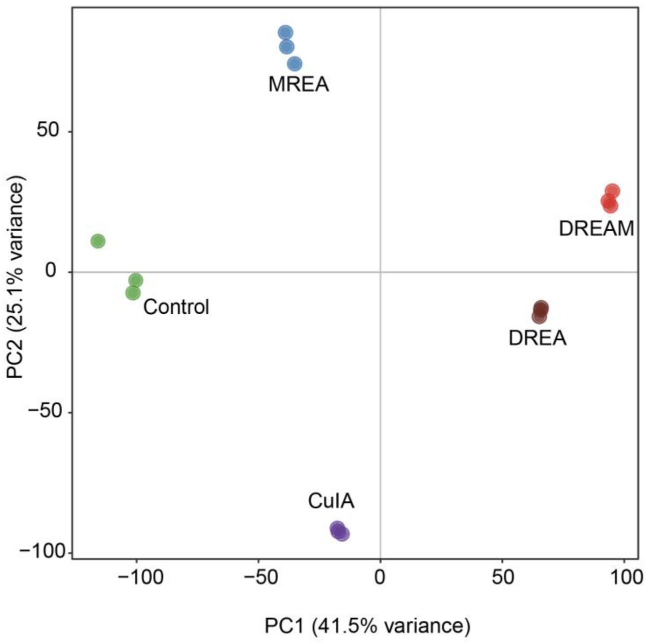

Figure S11. Principal component analysis (PCA) plot showed SCC7 cells with different treatments separated by their top three principal components.

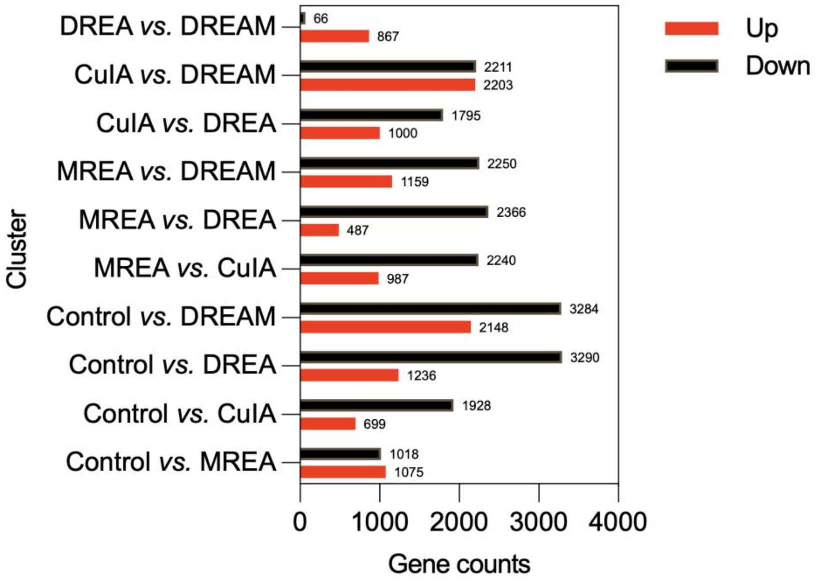

Figure S12. Differentially expressed gene (DEG) counts for each comparison cluster.

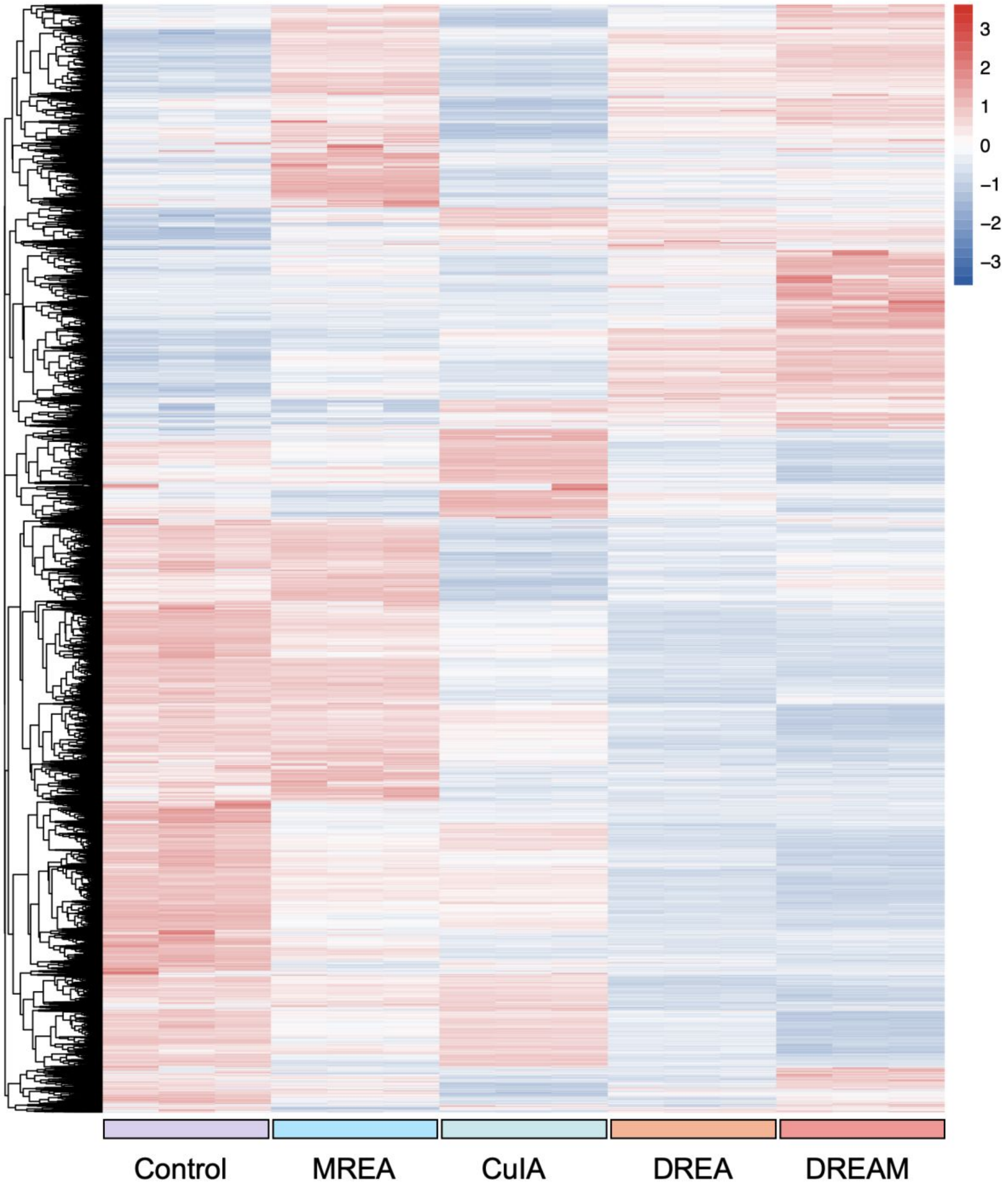

Figure S13. Heatmap of DEGs among SCC7 cells treated with PBS (Control), MREA, CuIA, DREA, and DREAM. The presented data comprises fragments per kilobase of exon per million fragments mapped (FPKM) values of genes in the sample, which have undergone Z-score normalization. The color scheme employed in the visualization denotes high-expression genes as red and low-expression genes as blue.

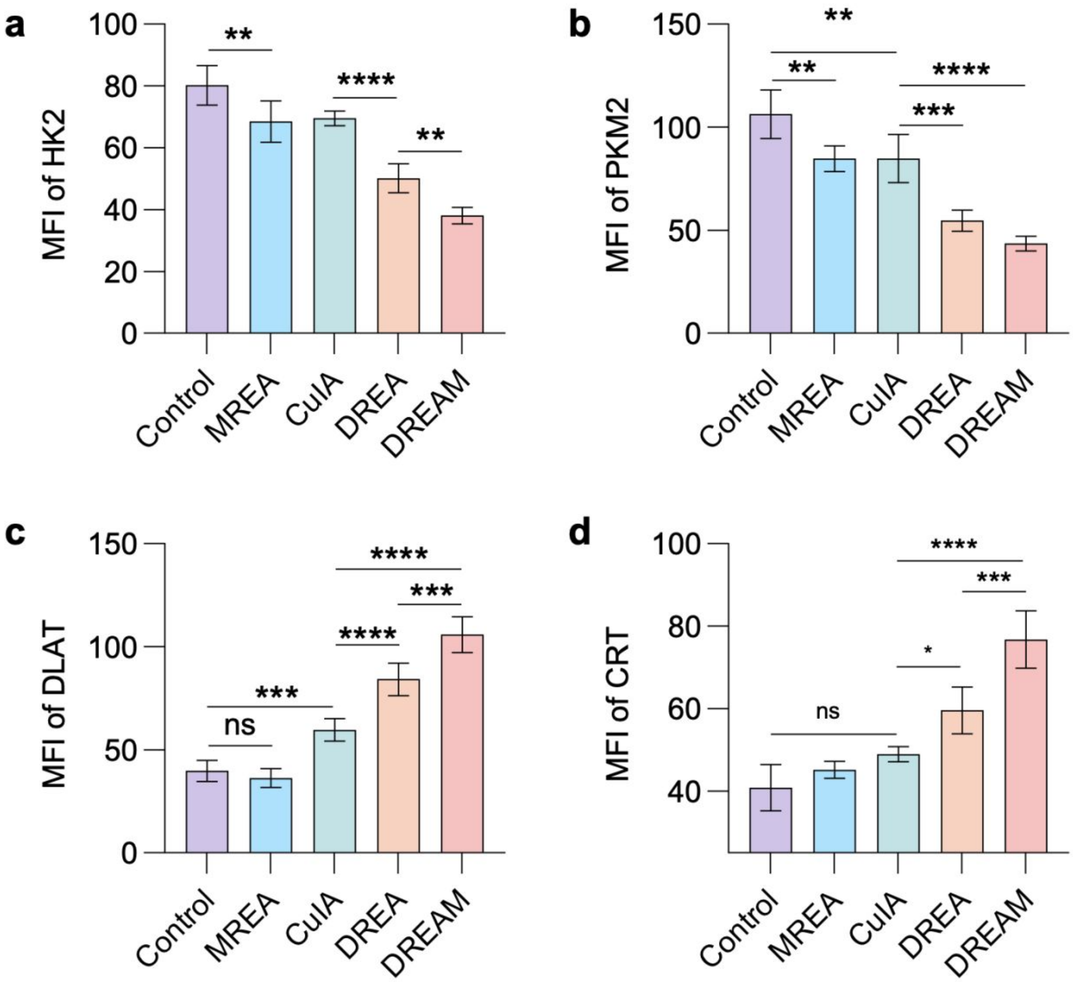

Figure S14. Quantitative analyses of the MFI of HK2, PKM2, DLAT, and CRT in SCC7 cells under different treatment conditions, as quantified using ImageJ based on CLSM images.

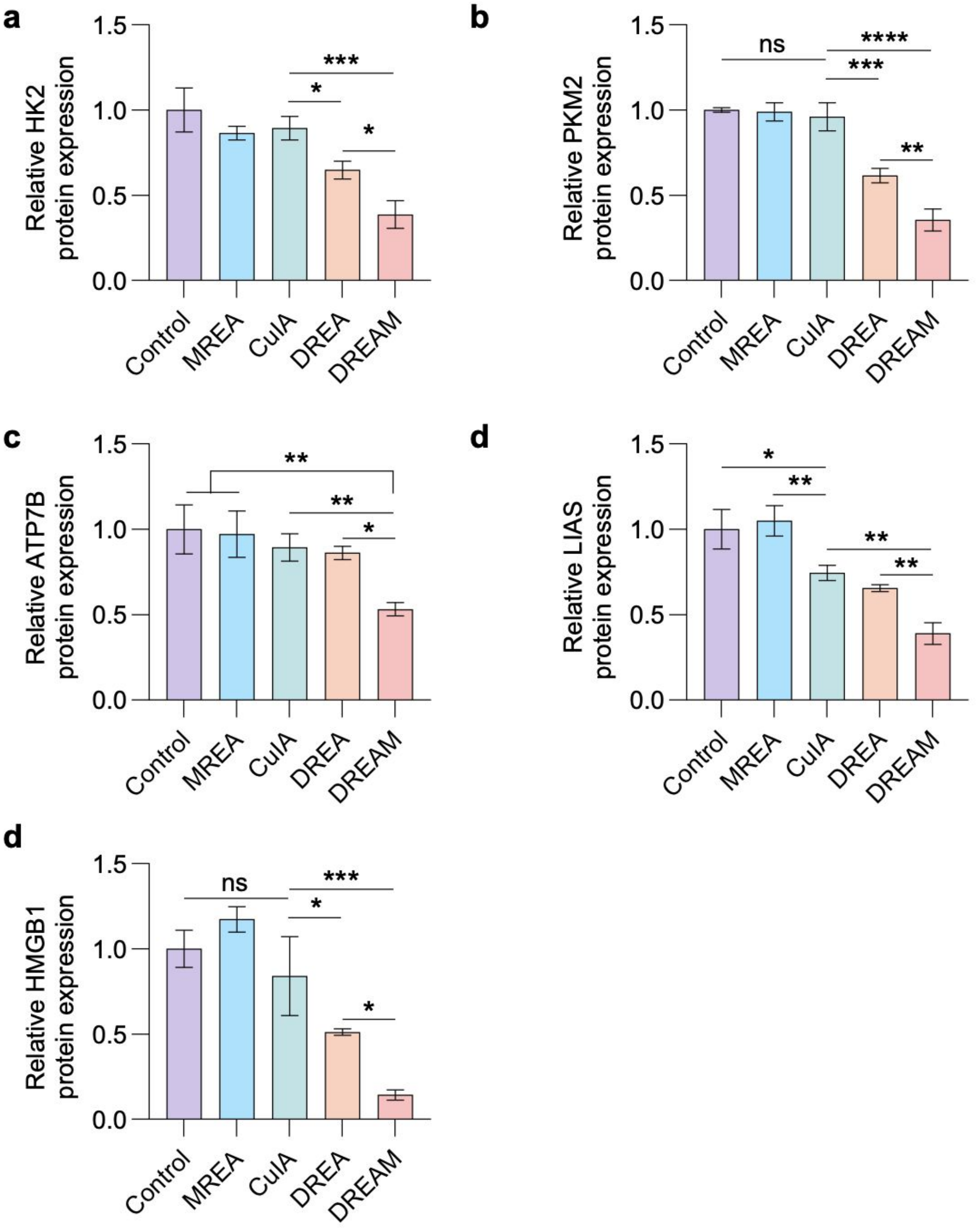

Figure S15. Quantitative analysis of the relative HK2, PKM2, ATP7B, LIAS, and HMGB1 protein expression in SCC7 cells treated with PBS (Control), MREA, CuIA, DREA, or DREAM for 24 hours, as quantified using ImageJ based on western blot data.

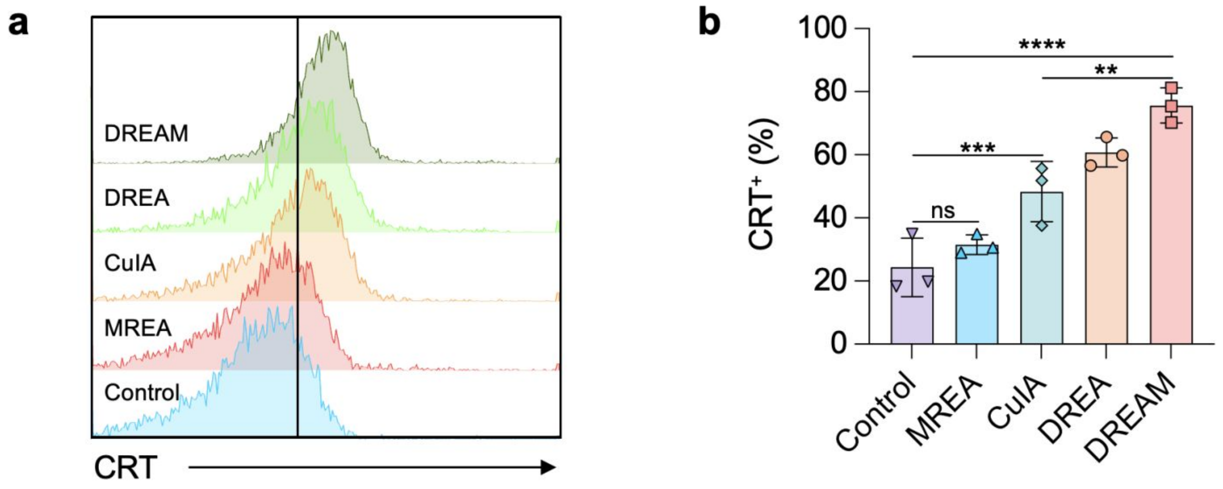

Figure S16. Representative FCM histograms (**a**) and quantitative analyses (**b**) of SCC7 cells expressing CRT on cell membrane.

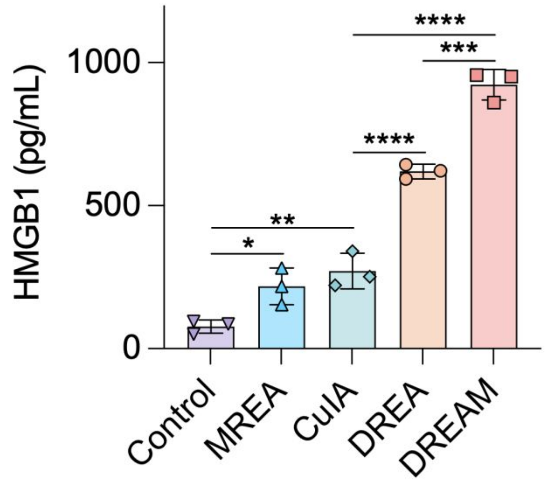

Figure S17. Released HMGB1 in the cell culture supernatant in SCC7 cells treated with PBS (Control), MREA, CuIA, DREA, or DREAM for 24 h, as quantified by enzyme-linked immunosorbent assay (ELISA).

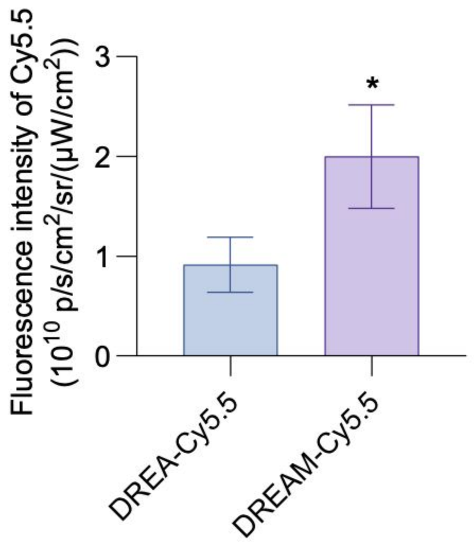

Figure S18. Quantification of the fluorescence intensity in the tumors from mice 24 hours after i.t. administration of DREA-Cy5.5 or DREAM-Cy5.5.

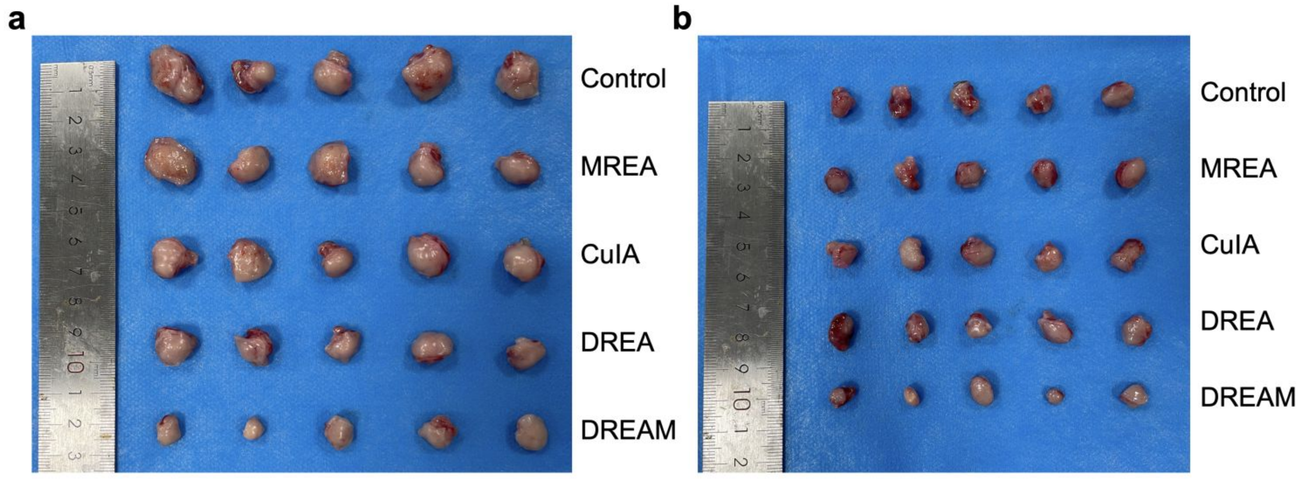

Figure S19. Tumor photographs of primary (a) and distant (b) SCC7 tumors with the indicated treatments (n = 5).

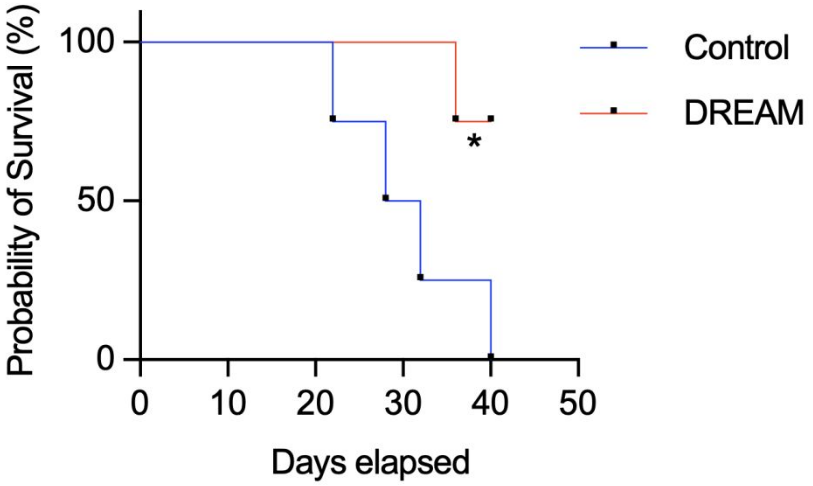

Figure S20. Survival curves of SCC7-bearing mice following the indicated treatments.

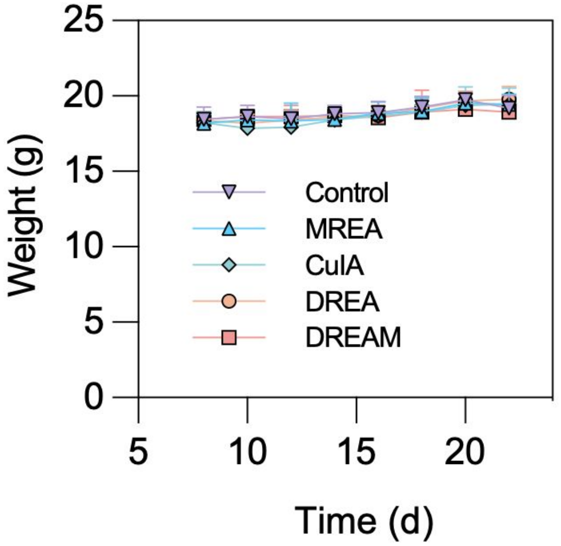

Figure S21. Mouse weight of the SCC7 tumor-bearing mice with the indicated treatments.

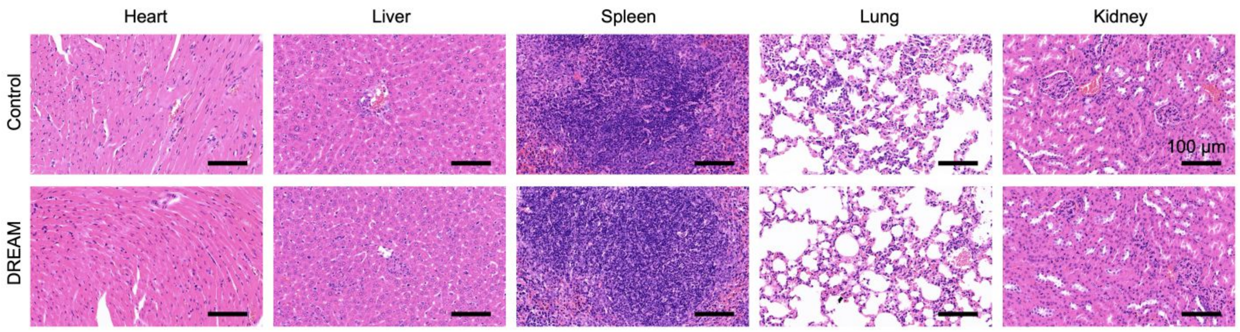

Figure S22. Hematoxylin and eosin (H&E) staining of major organs (heart, liver, spleen, lung, and kidney) from SCC7 tumor-bearing mice after treatment as indicated. Scale bars, 100 μm.

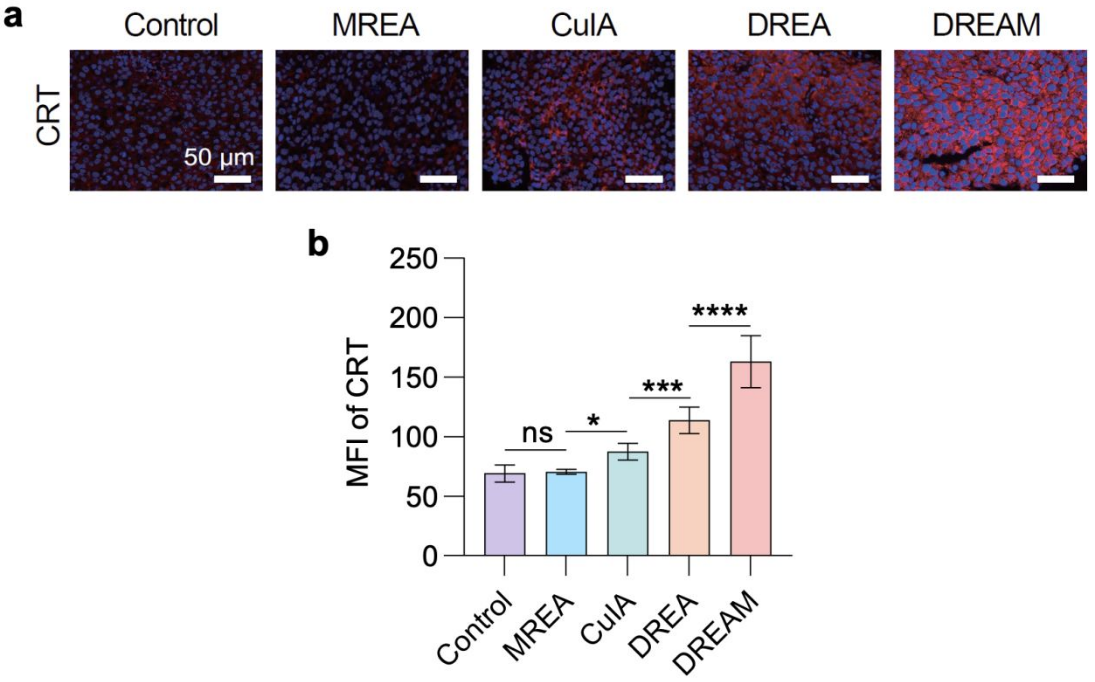

Figure S23. a) Representative immunofluorescent images showing SCC7 tumor sections were stained with the CRT (red) antibodies. Scale bars, 50 μm. **b)** Quantitative analyses of the MFI of CRT in tumor tissues harvested from SCC7 tumor-bearing mice.

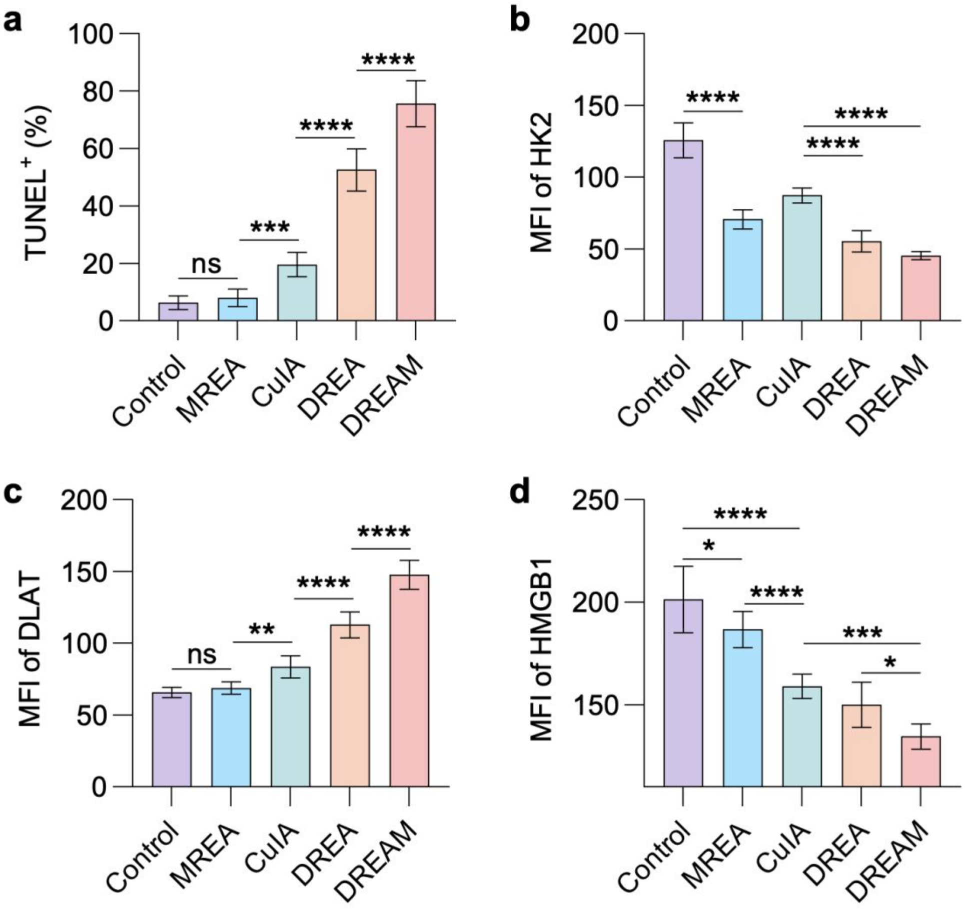

Figure S24. a) Percentage of TUNEL⁺ cells in tumor tissues harvested from SCC7 tumor-bearing mice. **b-d)** Quantitative analyses of the MFI of HK2(**b**), DLAT(**c**), and HMGB1(**d**) in tumor tissues harvested from SCC7 tumor-bearing mice.

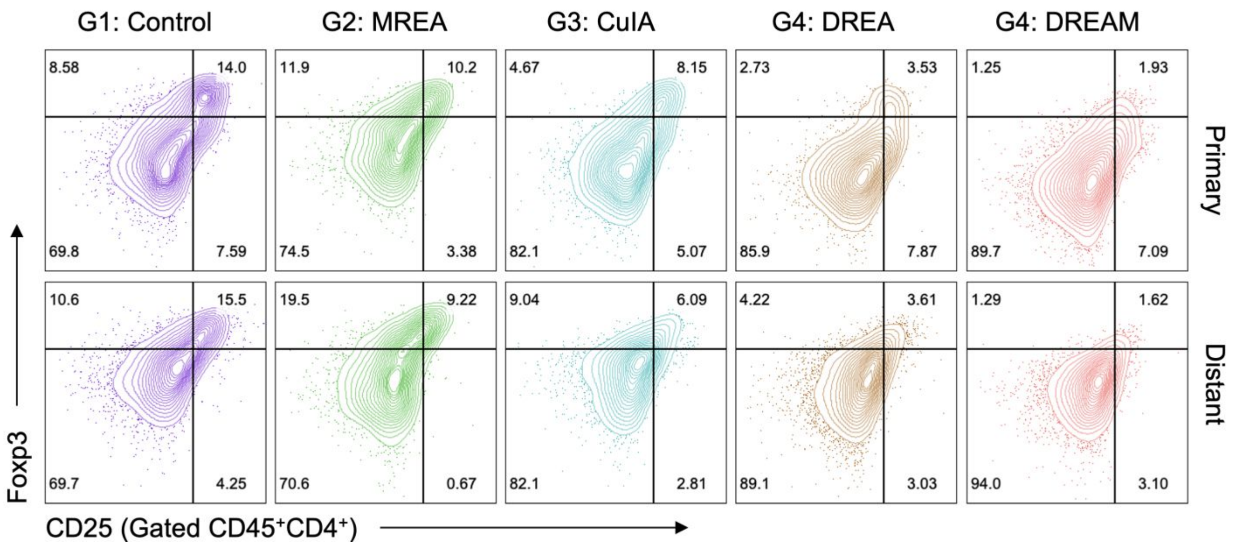

Figure S25. Representative FCM plots of Tregs ($CD45^+CD4^+CD25^+Foxp3^+$) in both primary and distant SCC7 tumors.

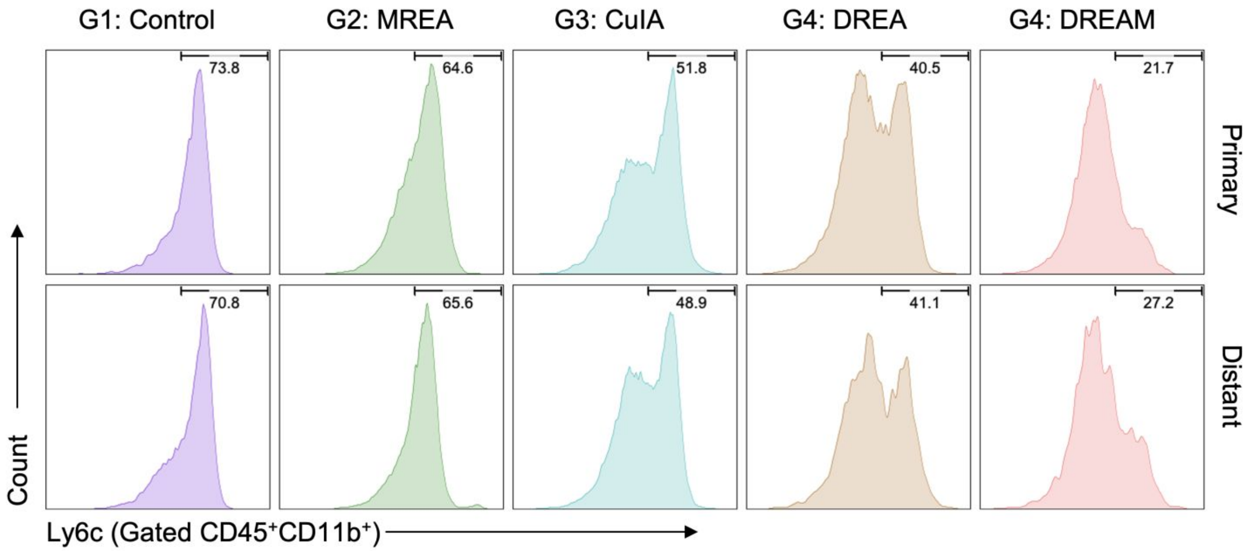

Figure S26. Representative FCM plots of M-MDSCs ($CD45^+CD11b^+Ly6c^+$) in both primary and distant SCC7 tumors.

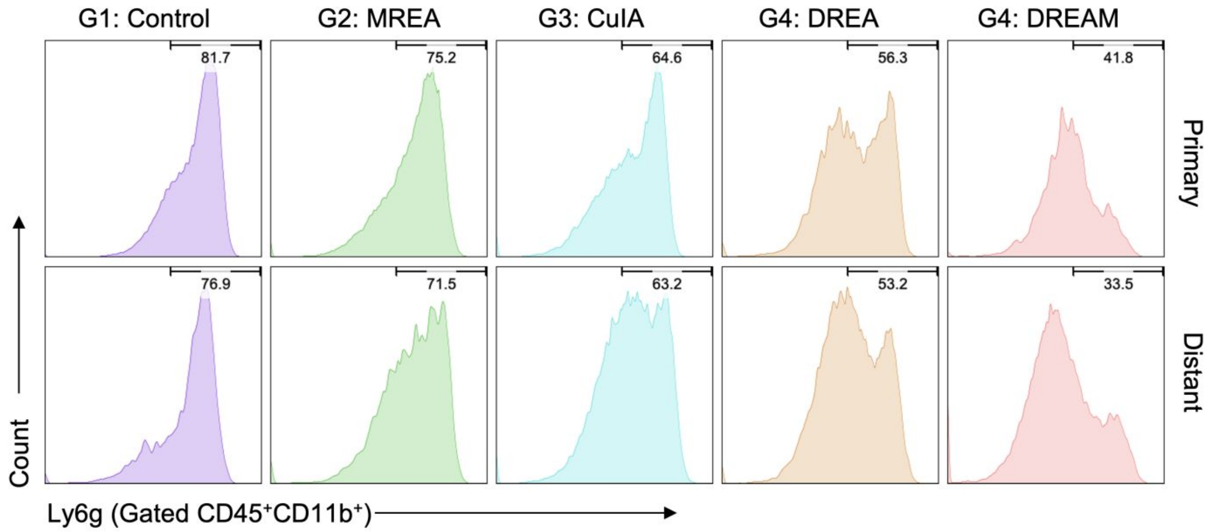

Figure S27. Representative FCM plots of PMN-MDSCs ($CD45^{+}CD11b^{+}Ly6g^{+}$) in both primary and distant SCC7 tumors.

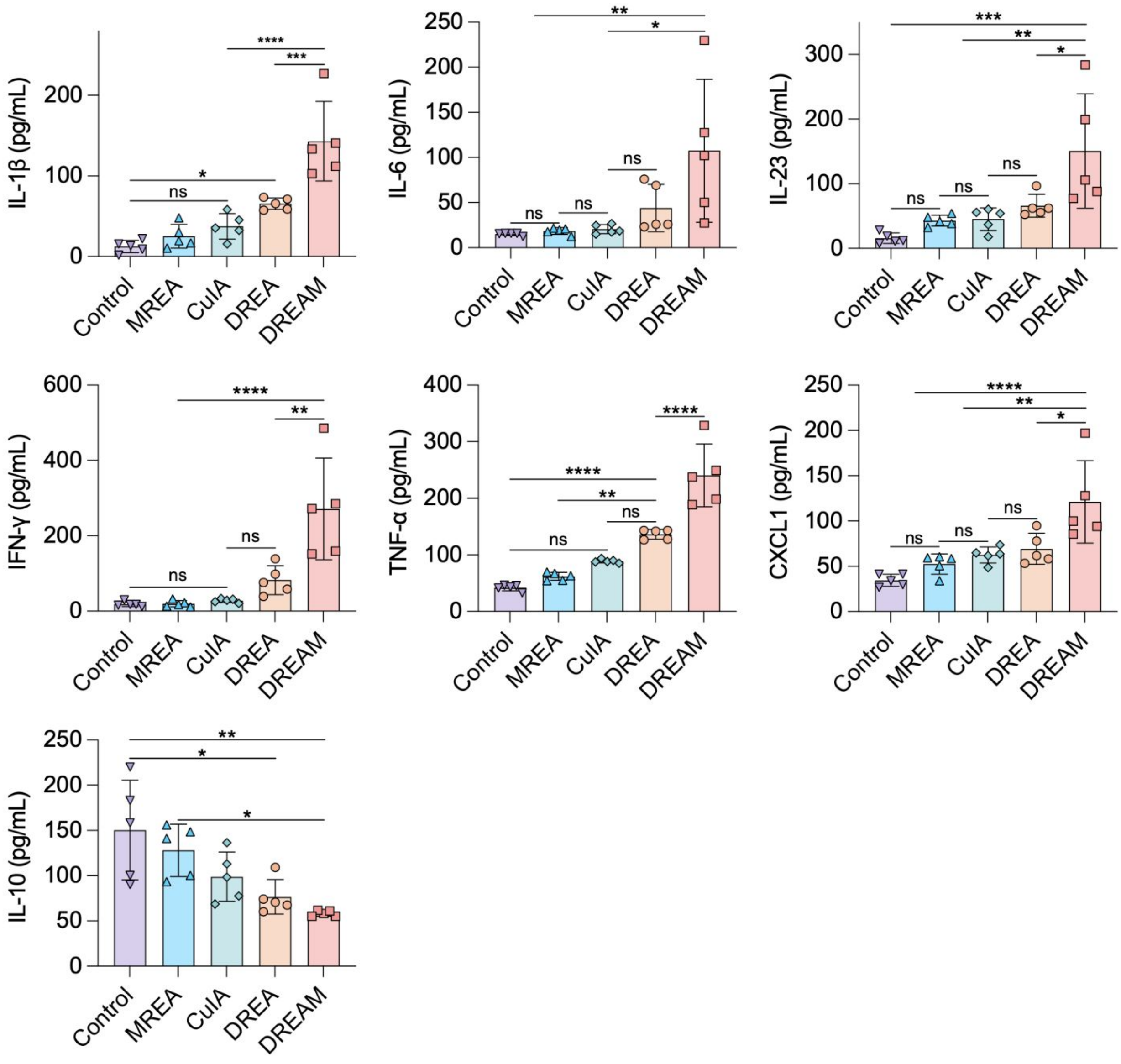

Figure S28. The concentrations of cytokines detected by cytometric bead array in the serum of tumor-bearing mice with different treatments.

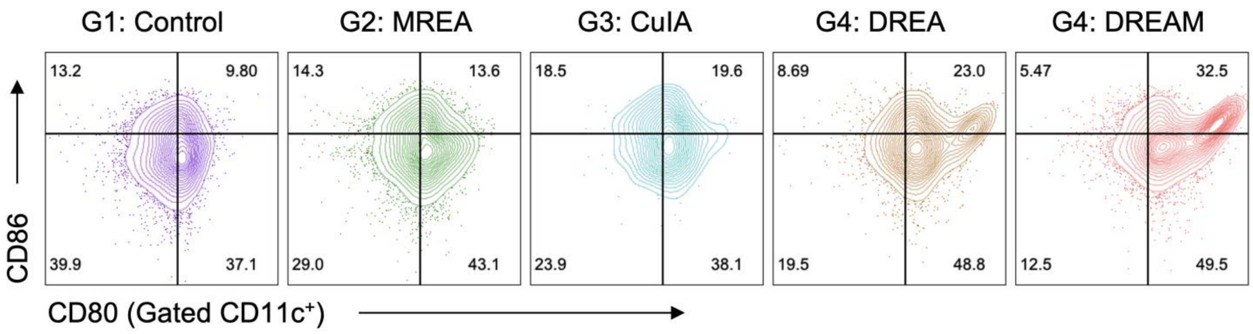

Figure S29. Representative FCM plots and the percentage of CD80$^+$CD86$^+$ DCs in tumor-draining lymph nodes (CD11c$^+$CD80$^+$CD86$^+$) of primary SCC7 tumors.

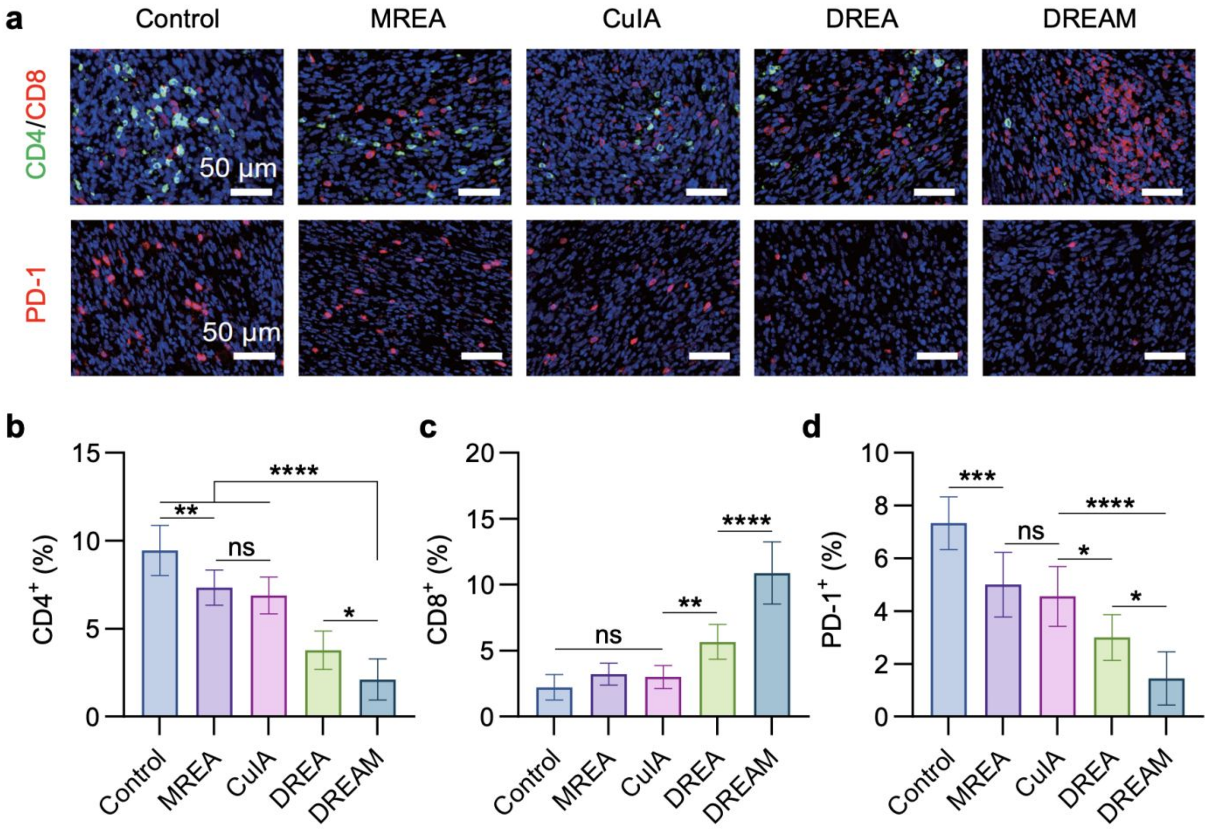

Figure S30. a) Representative immunofluorescent images showing SCC7 tumor sections were stained with the CD4 (green), CD8 (red), and PD-1 (red) antibodies. Scale bars, 50 µm. **b-d)**

Percentage of CD4$^+$ (**b**), CD8$^+$ (**c**), and PD-1$^+$ (**d**) T cells in tumor tissues harvested from SCC7 tumor-bearing mice.

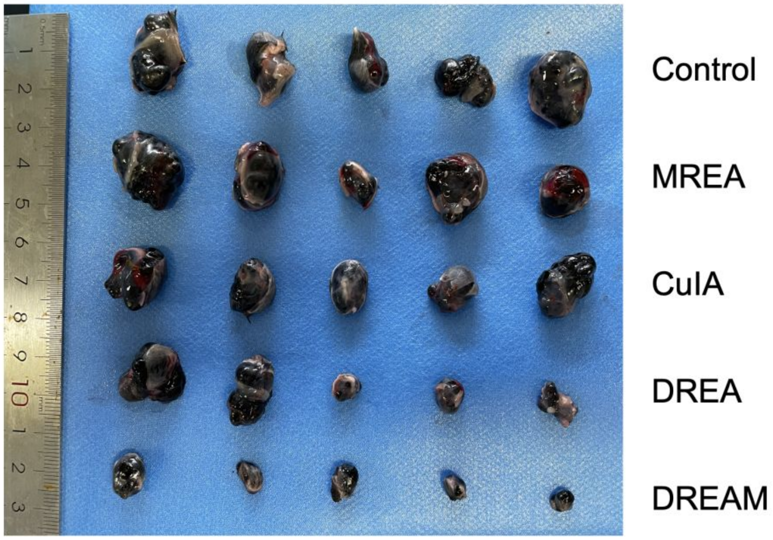

Figure S31. Tumor photographs of B16-OVA tumors with the indicated treatments.

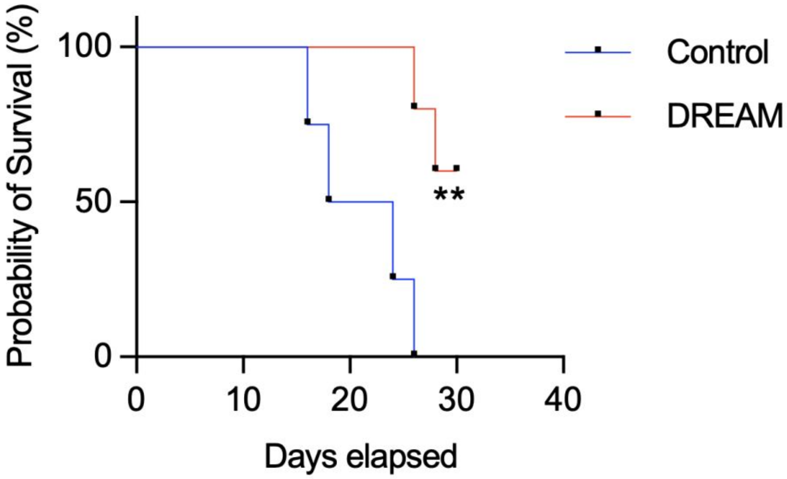

Figure S32. Survival curves of B16-OVA-bearing mice following the indicated treatments.

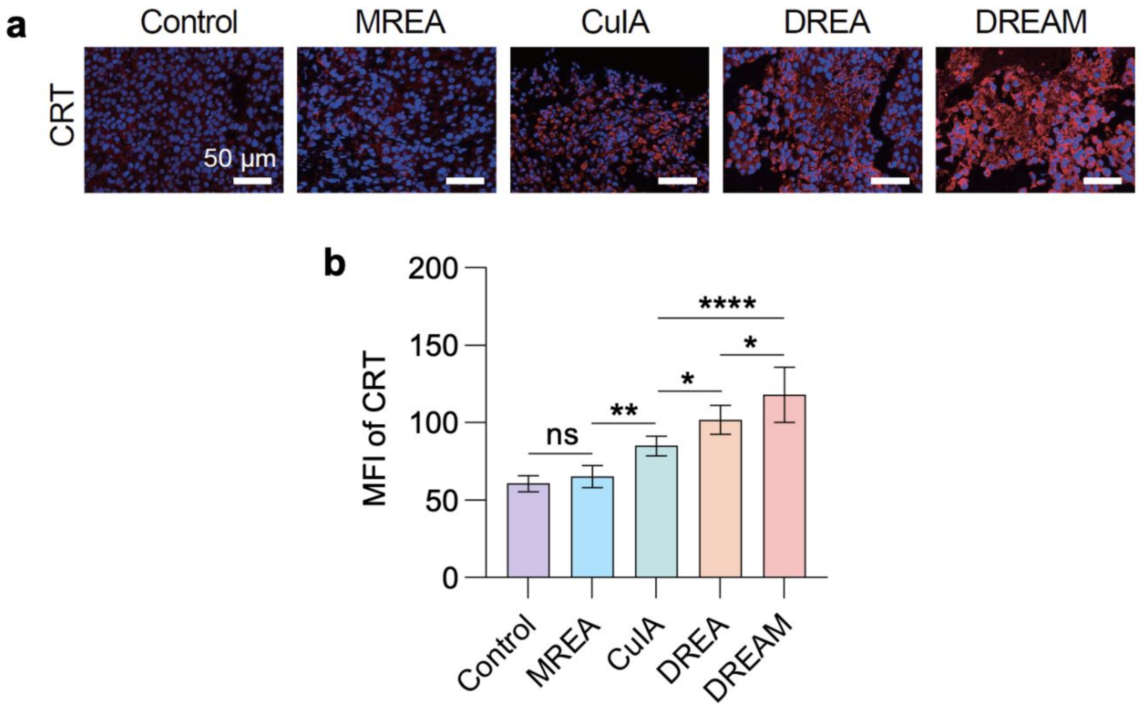

Figure S33. a) Representative immunofluorescent images showing B16-OVA tumor sections were stained with the CRT (red) antibodies. Scale bars, 50 μm. **b)** Quantitative analyses of the MFI of CRT in tumor tissues harvested from B16-OVA tumor-bearing mice.

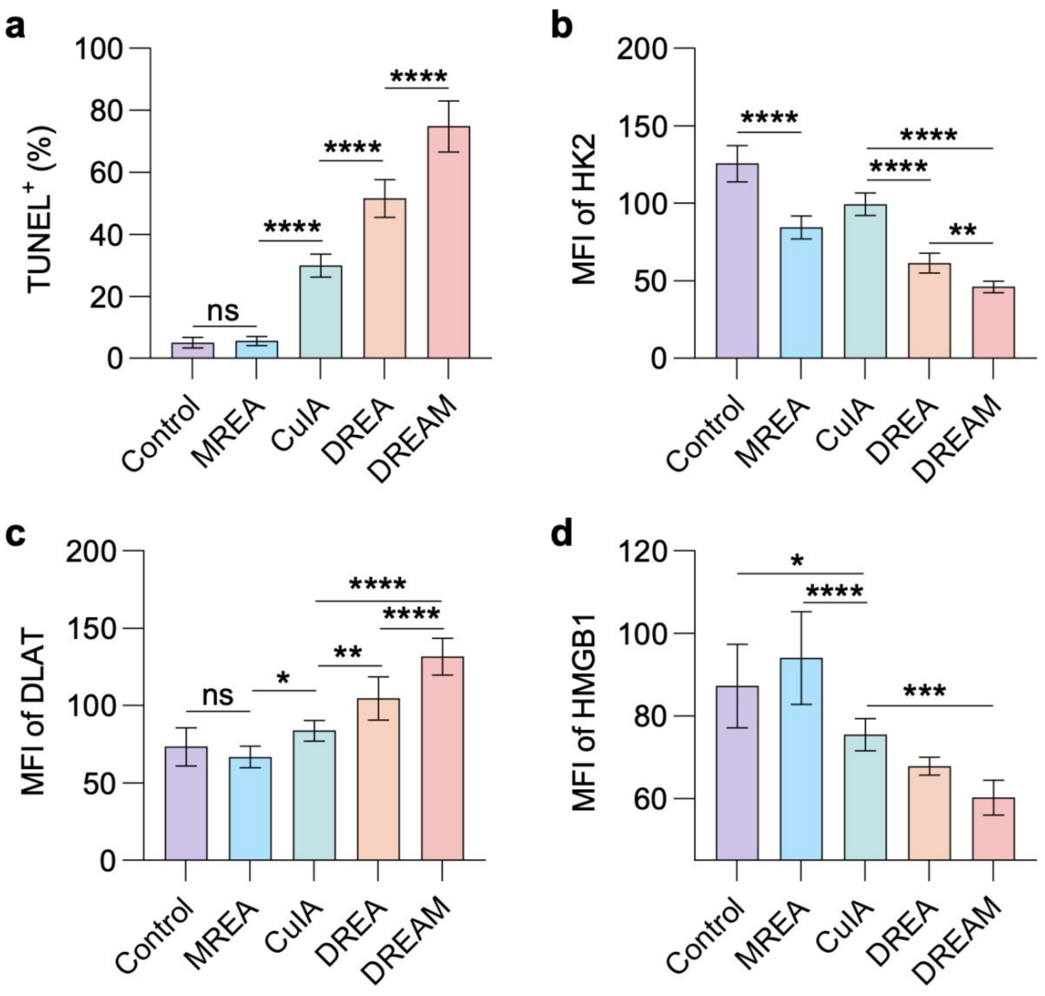

Figure S34. a) Percentage of TUNEL+ cells in tumor tissues harvested from B16-OVA tumor-bearing mice. **b-d)** Quantitative analyses of the MFI of HK2(**b**), DLAT(**c**), and HMGB1(**d**) in tumor tissues harvested from B16-OVA tumor-bearing mice.

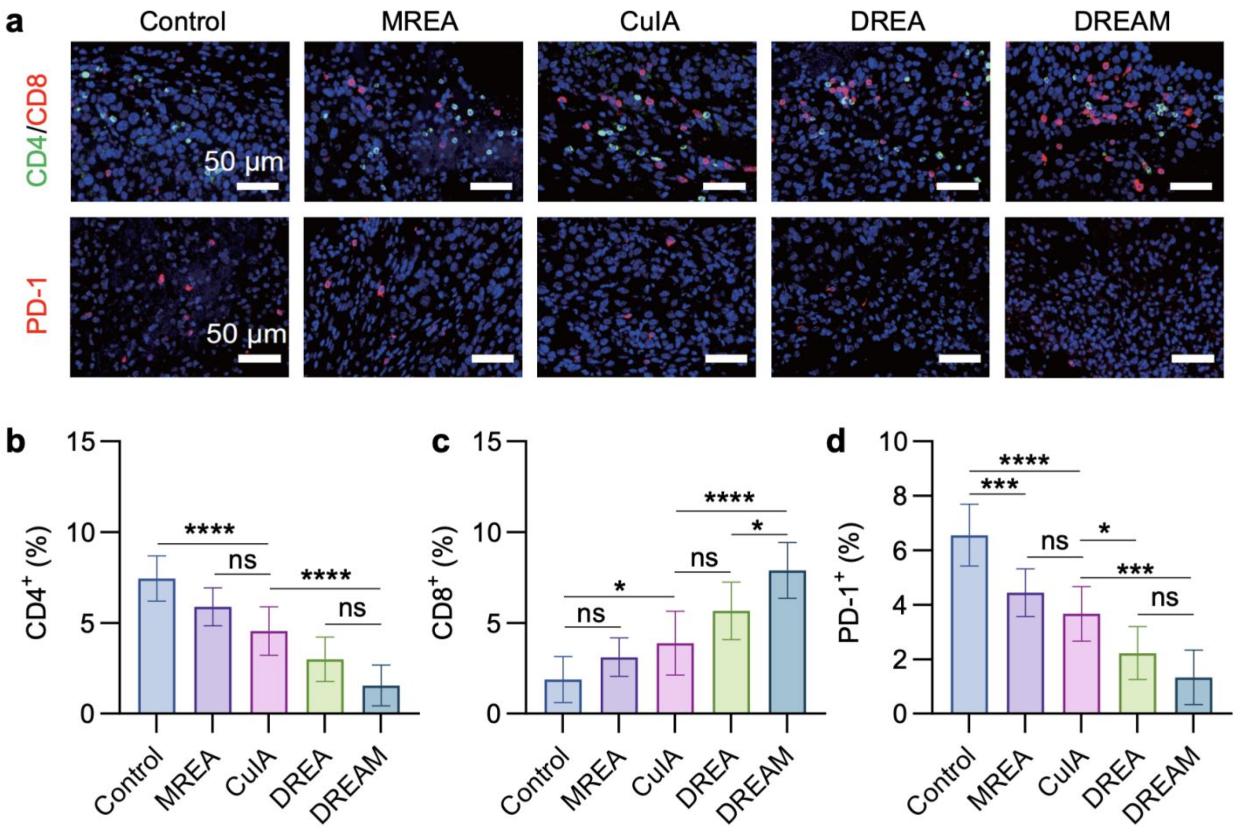

Figure S35. a) Representative immunofluorescent images showing B16-OVA tumor sections were stained with the CD4 (green), CD8 (red), and PD-1 (red) antibodies. Scale bars, 50 μm. **b-d)** Percentage of $CD4^{+}$ (**b**), $CD8^{+}$ (**c**), and $PD\text{-}1^{+}$ (**d**) T cells in tumor tissues harvested from B16-OVA tumor-bearing mice.

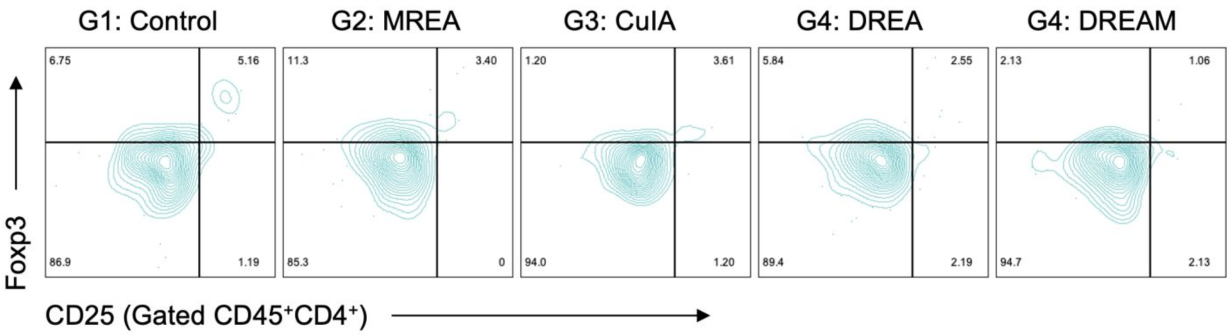

Figure S36. Representative FCM plots of Tregs ($CD45^+CD4^+CD25^+Foxp3^+$) in B16-OVA tumors.

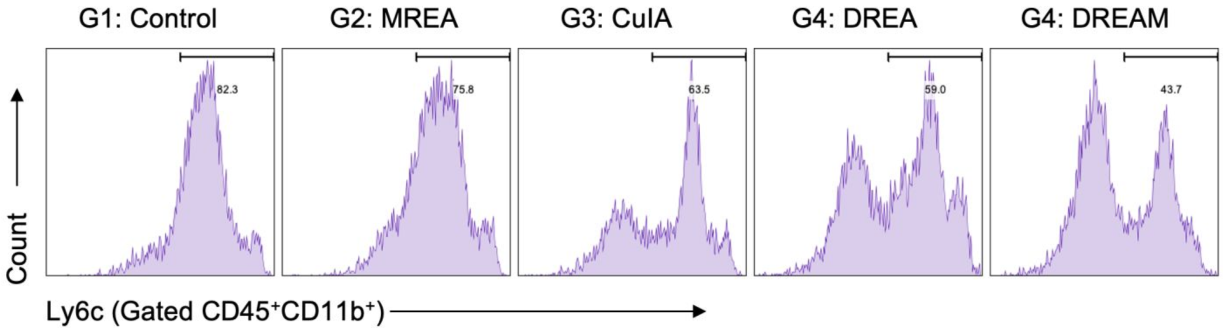

Figure S37. Representative FCM plots of M-MDSCs ($CD45^+CD11b^+Ly6c^+$) in B16-OVA tumors.

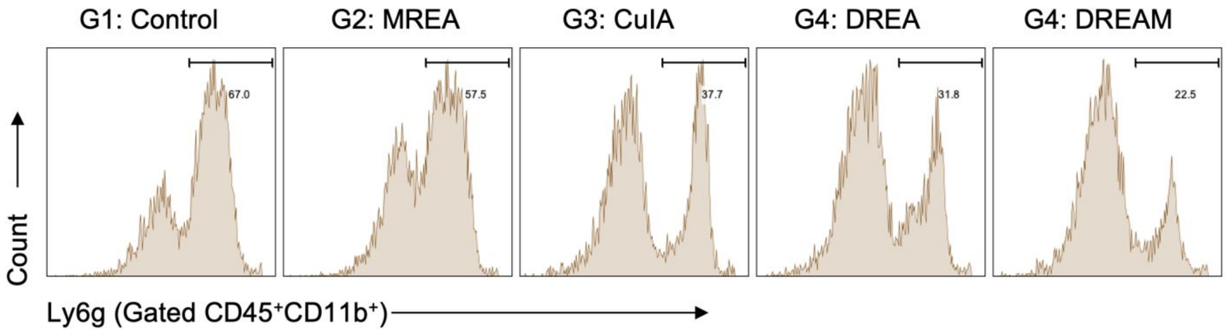

Figure S38. Representative FCM plots of PMN-MDSCs ($CD45^+CD11b^+Ly6g^+$) in B16-OVA tumors.

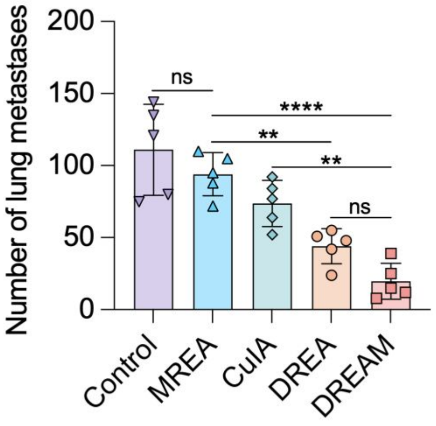

Figure S39. Numbers of lung metastatic nodules.

References

[1]. Martin, M., Cutadapt removes adapter sequences from high-throughput sequencing reads. *EMBnet.journal* **2011,** *17*, 10-12.

[2]. Kim, D.; Paggi, J. M.; Park, C.; Bennett, C.; Salzberg, S. L., Graph-based genome alignment and genotyping with HISAT2 and HISAT-genotype. *Nature Biotechnology* **2019,** *37* (8), 907-915.

[3]. Putri, G. H.; Anders, S.; Pyl, P. T.; Pimanda, J. E.; Zanini, F., Analysing high-throughput sequencing data in Python with HTSeq 2.0. *Bioinformatics* **2022,** *38* (10), 2943-2945.

[4]. Love, M. I.; Huber, W.; Anders, S., Moderated estimation of fold change and dispersion for RNA-seq data with DESeq2. *Genome Biology* **2014,** *15* (12), 550.

[5]. Sherman, B. T.; Hao, M.; Qiu, J.; Jiao, X.; Baseler, M. W.; Lane, H. C.; Imamichi, T.; Chang, W., DAVID: a web server for functional enrichment analysis and functional annotation of gene lists (2021 update). *Nucleic Acids Res* **2022,** *50* (W1), W216-W221.
